# Mathematical Modeling of Complement Pathway Dynamics for Target Validation and Selection of Drug Modalities for Complement Therapies

**DOI:** 10.3389/fphar.2022.855743

**Published:** 2022-04-19

**Authors:** Loveleena Bansal, Eva-Maria Nichols, Daniel P. Howsmon, Jessica Neisen, Christina M. Bessant, Fraser Cunningham, Sebastien Petit-Frere, Steve Ludbrook, Valeriu Damian

**Affiliations:** ^1^ Systems Modeling and Translational Biology, Computational Sciences, GSK, Upper Providence, Collegeville, PA, United States; ^2^ Immunology Research Unit, GSK, Stevenage, United Kingdom; ^3^ Department of Chemical and Biological Engineering, Rensselaer Polytechnic Institute, Troy, NY, United States

**Keywords:** complement pathway, mathematical modeling, Quantitative Systems Pharmacology, drug modality selection, target validation, dose prediction

## Abstract

**Motivation:** The complement pathway plays a critical role in innate immune defense against infections. Dysregulation between activation and regulation of the complement pathway is widely known to contribute to several diseases. Nevertheless, very few drugs that target complement proteins have made it to the final regulatory approval because of factors such as high concentrations and dosing requirements for complement proteins and serious side effects from complement inhibition.

**Methods:** A quantitative systems pharmacology (QSP) model of the complement pathway has been developed to evaluate potential drug targets to inhibit complement activation in autoimmune diseases. The model describes complement activation *via* the alternative and terminal pathways as well as the dynamics of several regulatory proteins. The QSP model has been used to evaluate the effect of inhibiting complement targets on reducing pathway activation caused by deficiency in factor H and CD59. The model also informed the feasibility of developing small-molecule or large-molecule antibody drugs by predicting the drug dosing and affinity requirements for potential complement targets.

**Results:** Inhibition of several complement proteins was predicted to lead to a significant reduction in complement activation and cell lysis. The complement proteins that are present in very high concentrations or have high turnover rates (C3, factor B, factor D, and C6) were predicted to be challenging to engage with feasible doses of large-molecule antibody compounds (≤20 mg/kg). Alternatively, complement fragments that have a short half-life (C3b, C3bB, and C3bBb) were predicted to be challenging or infeasible to engage with small-molecule compounds because of high drug affinity requirements (>1 nM) for the inhibition of downstream processes. The drug affinity requirements for disease severity reduction were predicted to differ more than one to two orders of magnitude than affinities needed for the conventional 90% target engagement (TE) for several proteins. Thus, the QSP model analyses indicate the importance for accounting for TE requirements for achieving reduction in disease severity endpoints during the lead optimization stage.

## 1 Introduction

The complement system forms an important component of innate immunity by providing the first line of defense against infections. It is composed of several proteins produced mainly by the liver or expressed on the surface of the cells. The complement proteins participate in a cascade that forms large protein complexes on the cell surface, opsonizing and killing the target cell. The tight regulation of the pathway through various regulatory proteins and cell surface receptors protects the host cells against complement-mediated cell death, and there exists a fine balance between complement activation and regulation. However, dysregulation of the complement pathway due to protein deficiencies or genetic mutations can unfavorably tip the balance (Morgan and Harris, 2015) toward over-activation, leading to autoimmune diseases or under-activation increasing susceptibility to infections.

The complement system is widely known to contribute to the pathology of several diseases and is an active area of drug development. However, despite a wealth of knowledge on the complement cascade and its components, very few drugs have made it to the final regulatory approval (Harris, 2018). The presence of multiple regulatory and activating proteins, the high abundance and turnover rate of most complement proteins, as well as serious side effects from complement inhibition are just some of the emerging factors that make drug development extremely challenging for therapies targeting complement proteins. Thus, there is a critical need for the investigation of efficacious and cost-effective complement modulatory therapies for patients.

Quantitative systems pharmacology (QSP) is an *in silico* modeling approach that combines the knowledge of disease processes with drug mechanisms to evaluate targets and drug candidates for modulating disease severity and biological pathways (Sorger et al., 2011). It is now widely used in the pharmaceutical industry to estimate drug efficacy, clinical doses, biomarker responses, and patient stratification (Nijsen et al., 2018). To take a step toward overcoming the challenges in developing drugs targeting the complement pathway, we have developed a comprehensive QSP model describing complement activation *in vivo* in humans *via* alternative and terminal pathways. The model contains several variables representing complement proteins, protein complexes, cleavage products, and intermediates participating in biochemical processes across the plasma and surface of host cells.

There have been a few previous reports of mathematical modeling for the complement pathway. The previous models have lacked sufficient details for a comprehensive description of the pathway or translation of modeling results from *in vitro* to *in vivo* activation of the complement system in humans. The first attempts at mathematical descriptions of the pathway included simplified descriptions of the complex dynamics in key aspects of complement activation such as the amplification loop (Reeve and Woo, 1982) in the alternative pathway and formation of the membrane attack complex (MAC) (Hirayama et al., 1996). Korotaevskiy et al. (2009) developed a combined mathematical model of classical, alternative, and terminal pathways of the complement system; however, the impact of complement regulators and the differentiation between pathway activation on the cell surface *versus* in the plasma were not included. Over the years, more comprehensive models have been developed (Zewde et al., 2016; Zewde and Morikis, 2018; Tille et al., 2020; Zewde et al., 2021) with descriptions of the plasma and cell surface regulators. However, these models have included simplified descriptions of the dynamics of regulators such as Properdin, clusterin, and vitronectin which do not capture all regulatory effects. In a recent modeling assessment (Zewde et al., 2021), an integrated systems biology model for all the three complement pathways, alternative, classical, and lectin, has also been developed for modeling the complement response against pathogens such as N. meningitidis. The focus in this study is on evaluating target proteins in the alternative and terminal pathways with a mechanistic description of complement over-activation observed in autoimmune diseases. Moreover, the previous modeling assessments have not evaluated the effect of several complement proteins as potential targets for drug development, and the evaluation of drug dosing requirements for human clinical trials has also not been conducted.

The computational complement pathway model or simply the “complement model” or “QSP model” developed in this study comprehensively describes the dynamics of complement activation through the alternative and terminal pathways. The tick-over and amplification of the alternative pathway have been described in plasma and on the surface of erythrocytes, respectively. An *in silico* description of the lysis of host cells due to complement over-activation has been modeled due to the formation of the MAC *via* the terminal pathway. The complement model also includes an expanded and more physiologically relevant description of the plasma and cell surface regulatory proteins such as Properdin or factor P (FP), clusterin, vitronectin, CD59, complement receptor type 1 (CR1), CD55 or decay-accelerating factor (DAF), factor H (FH), and factor I (FI). For refining the model dynamics and estimating the kinetic parameters, several published and in-house *in vitro* datasets were used for validation of the dynamics of alternative and terminal pathways.

The model simulations, *via* reduction in FH and CD59, reflect the mechanistic basis of complement activation in diseases such as atypical hemolytic uremic syndrome (aHUS) and paroxysmal nocturnal hemoglobinuria (PNH). These fundamental representations of simulated disease states have been used for evaluating the effect of potential complement targets on reducing disease severity *via* downstream biomarkers (e.g., C3a, C5a, and MAC) and the lysis of erythrocytes.

A number of drug candidates are currently in development across the pharmaceutical industry (Harris, 2018; Mastellos et al., 2019; Zelek et al., 2019) for targeting complement proteins. Several complement proteins such as C3, factor B (FB), factor D (FD), C5, C6, and C7 are present in the blood at extremely high concentrations (∼mg/ml) and/or have fast turnover rates (Harris, 2018) as compared to most other targets in inflammatory diseases, such as cytokines (∼pg/ml). This poses challenges around the feasibility of dosing enough for drugs to engage the target and risk of potential side effects due to it. Thus, we have evaluated drug doses needed for small-molecule and large-molecule antibody drugs to engage complement proteins to provide “dosing tractability” estimates within feasible dose ranges of these two modalities.

There are also short-lived intermediate proteins in the complement pathway (e.g., C3b, C3bB, and C3/C5 convertases) formed due to proteolytic cleavage and conformational changes. These cleavage products have not been fully explored in the pharmaceutical industry but represent promising drug targets due to their low concentrations and the potential of engaging them with lower drug doses. Therefore, an assessment of the dosing tractability was performed for these novel targets as well. Additionally, since higher doses of drug candidates may be compensated by lower, that is, more potent drug affinities to attain the required level of target engagement and *vice versa*, an assessment of both the dose levels and drug affinities for target engagement has been done. These evaluations have been used to guide the drug development for complement therapeutics during early target validation and lead optimization phases to support assessments such as modality selection, dosing tractability, and effects of target engagement on complement pathway endpoints.

In Section 2, the development of the complement pathway model with alternative and terminal pathways as well as the dynamics of regulatory proteins has been described. This section also describes the methods for preclinical *in vitro* assays that were used for validating the QSP model dynamics. Section 3 shows the simulation results for the complement model starting with the comparisons with preclinical data. After validation of the pathway dynamics with *in vitro* data, the model has been further validated by predicting the effect of deficiency in regulatory proteins on the pathway activation *in vivo* in humans which have been observed in several complement-driven disease pathologies. Furthermore, an analysis of the effect of inhibiting potential drug targets on reducing complement activation is shown to identify promising targets for drug development. In addition to reducing the disease severity, the tractability of the targets due to their drug dosing and affinity requirements has been analyzed to provide a comprehensive evaluation of developing drugs targeting complement proteins and intermediates. Section 4 discusses the key results obtained in this work, the scope of the analysis conducted, model limitations, and thoughts on further explorations for future work using the computational modeling approach.

## 2 Methods

### 2.1 Mathematical Model Development

**FIGURE 1:**
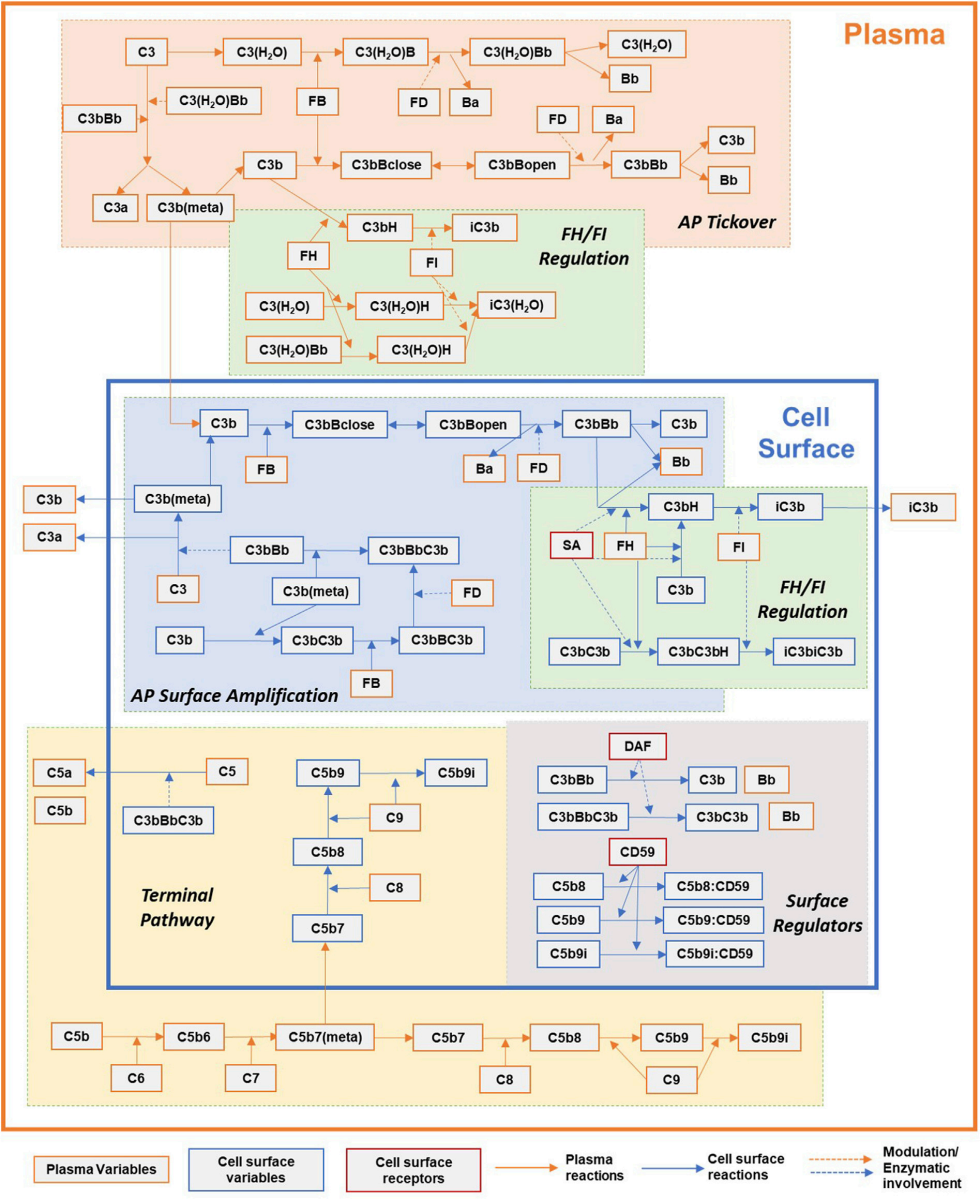
Simplified overview of the processes in the complement pathway model for plasma and cell surface reactions. Key processes shown here: alternative pathway (AP) tickover, regulation by FH and FI in plasma and cell surface, AP surface amplification, terminal pathway, and surface regulators: sialic acid, DAF, and CD59. Dynamics for binding of complement proteins to Properdin are not included here for simplicity and represented separately in Figure 2. Other key processes included in the model but not shown here: dynamics of regulation by CR1, clusterin, and vitronectin.

The complement model describes pathway activation in plasma and on the surface of erythrocytes *via* the alternative and terminal pathways. The model consists of 311 state variables and 139 kinetic parameters. The processes included in the computational model are detailed in the following sections and presented in Figure 1. A summary of the model variables, reactions, parameter values, their sources, and ordinary differential equations (ODEs) is provided in supplementary files. The reaction fluxes have been derived using mass action kinetics for most processes and Michaelis–Menten kinetics for enzymatic reactions. All the model development and simulation work have been carried out using the SimBiology toolbox in MATLAB 2019a (Mathworks).

#### 2.1.1 Alternative Pathway and Its Regulators

##### 2.1.1.1 Tickover reactions

The tickover reactions in the alternative pathway provide a sustained low level of complement activation in plasma which can get amplified if a pathogen or cell surface is detected by the complement proteins. The alternative pathway is initiated by the spontaneous hydrolysis of the thioester in C3, forming C3(H_2_O) in the plasma or “fluid” phase. C3(H_2_O) can then bind with FB, generating C3(H_2_O)B. There are conflicting reports (Pryzdial and Isenman, 1988; Bexborn et al., 2008) regarding the relative affinity of FB for C3(H_2_O) and C3b. Because of this discrepancy, the model assumes that C3(H_2_O) and C3b bind with FB with the same binding rate constants. FD cleaves C3(H_2_O)B producing the tickover convertases C3(H_2_O)Bb and Ba. Once activated, C3(H_2_O)Bb can cleave C3, producing C3a and C3b with a reactive thioester denoted as C3b(meta) in the model. The kinetic efficiency of the fluid convertase C3(H_2_O)Bb is assumed to be half of C3bBb (based on (Pangburn and Müller-Eberhard, 1986; Bexborn et al., 2008)). Since studies on particle-bound and cobra venom factor convertases indicate that the non-catalytic subunit of the convertase only affects its $K_{m}$ (Tille et al., 2020), the model uses the $k_{cat}$ reported for C3bBb and modifies the $K_{m}$ to match experimental observations for the kinetic efficiency.

The metastable thioester of C3b(meta) either binds to the cell surface or gets inactivated in plasma. If the metastable thioester decays before it binds to the cell surface, it can still participate in the fluid-phase reactions described for C3(H_2_O) by first binding with FB and then FH (Volanakis, 1990). The products of these reactions are cleaved by FD and FI. Surface plasmon resonance (SPR) studies of C3b binding to FB have revealed a two-state conformational change model (Harris et al., 2005; Hourcade and Mitchell, 2011) for the proconvertase C3bB which has been adopted in the model for both plasma and cell surface reactions. C3b binds with FB to form a “closed C3bB,” denoted as C3bBclose, which undergoes a rapid conformation change to form C3bBopen (following the notation in (Torreira et al., 2009)) which is then cleaved by FD to form C3bBb. The parameters for this conformation change are assumed to be the average of the parameter values estimated from multiple studies (Hourcade and Mitchell, 2011). The fluid-phase C3bBb can cleave C3, generating more C3a and C3b(meta) in the surrounding fluid. The C3-convertase is very unstable, and the Bb domain can irreversibly disassociate with a half-life of approximately 1.5 min (Pangburn and Müller-Eberhard, 1986). The isolated Bb has been shown to lose 99% of its activity toward C3 after decay of the C3-convertase (Fishelson and Müller-Eberhard, 1984); thus, the model assumes that Bb does not participate in any other reactions in the complement cascade.

##### 2.1.1.2 Regulation by Factor H and Factor I

In plasma, C3(H_2_O) can bind to FH, generating C3(H_2_O)H. This represents the first point of regulation in the alternative pathway as the binding to FH competes with the binding to FB and further activation. FI can cleave C3(H_2_O)H, releasing FH and the inactive product iC3(H_2_O). FH also binds with C3b, creating the complex C3bH in the fluid and on the cell surface. The model assumes that FH binds to C3b with a slightly higher affinity than that of C3(H_2_O) based on the parameter fitting with internal data (data not shown). Unlike the FH reactions in the fluid phase, the cell surface reaction depends on the quality of the surface, usually attributed to the presence of sialic acid (SA) residues, and studies have shown (Kazatchkine et al., 1979) that the affinity of FH for C3b in the absence of SA is significantly lower, which has been accounted by the model by adding a linear effect of SA residues on FH binding with C3b. FI can cleave C3b to iC3b in the presence of a cofactor such as FH or CR1, and then subsequently to C3c and C3dg.

##### 2.1.1.3 Surface Amplification

Amplification of the alternative pathway can occur on suitable cell surfaces. Although the thioester of C3b appears to have little preference for various cell surface moieties (Müller-Eberhard, 1988), regulation by soluble FH and membrane-bound receptors classify cells as activators or non-activators of the alternative pathway. Cell surface-bound C3b occupies binding sites denoted as C34b* in the model. As in the fluid phase, surface-bound C3b interacts with fluid-phase FB to form the proconvertase closed and open confirmations, and then with FD to form the C3-convertase C3bBb. Cell surface C3-convertase is also assumed to be unstable with a half-life of 1.5 min as modeled for the fluid phase.

On the surface, C3bBb further cleaves C3 and perpetuates the amplification loop of the alternative pathway. However, since the convertase is bound to the surface, the extremely labile C3b(meta) generated near the surface has a higher probability of binding that surface. C3b(meta) also interacts with C3bBb to form the C5-convertase, C3bBbC3b. Furthermore, C3b(meta) can bind to the α-chain of the surface-bound C3b (Hong et al., 1991, p. 199), generating C3b dimers on the surface. C3b dimers are also assumed to interact with fluid-phase FB and FD to form surface-bound C5-convertase.

##### 2.1.1.4 Role of Properdin

Properdin or factor P (FP) is the only known positive regulator of the alternative pathway. In plasma, FP exists as a mixture of head-to-tail dimers, trimers, and tetramers (Smith et al., 1984). It is assumed that the Properdin oligomers provide two, three, and four binding sites, respectively, on dimers, trimers, and tetramers for C3b binding and *de novo* assembly of the C3-convertase. The quantitative implementation for the role of Properdin in the activation of the alternative pathway is based on the model proposed by Hourcade (2006). In addition to the stabilization of the C3 and C5-convertase, Properdin promotes the association of C3b to FB, binds to the surface-bound C3b or other ligands, and uses its unoccupied binding sites as receptors for nascent C3b and preformed C3-proconvertase and convertase. The binding of Properdin to the surface of erythrocytes is assumed to be fully dependent on the initial deposition of C3b in line with recent studies (Harboe et al., 2004), and its controversial role as a pattern-recognition molecule with direct binding to cell surfaces is not taken into account in the model.

**FIGURE 2:**
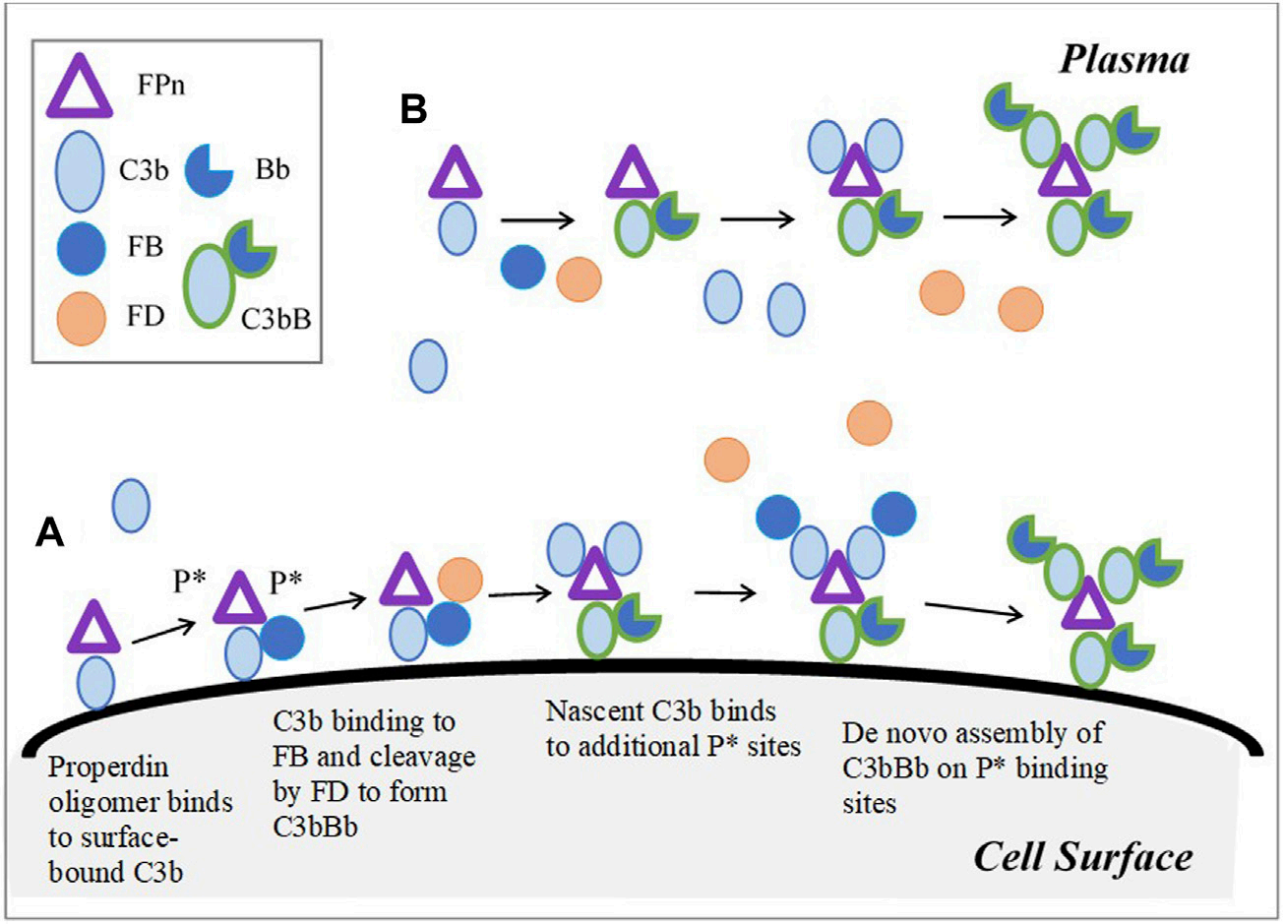
Model implementation for Properdin dynamics in plasma and cell surface (expanded based on Figure 6 in Hourcade (2006)). **(A)** Cell surface: Properdin binds to surface bound C3b and C3-proconvertases, C- convertase, or iC3b, and provides two additional sites P* for the *de novo* assembly of the convertase. FPn binding to only surface-bound C3b is shown in the figure for simplicity. Similarly, in addition to C3b-P* binding, C3 proconvertase–C3bB, C3b-convertase, and iC3b can occupy P* sites. **(B)** Plasma: C3b or its complexes and cleavage products bind to three binding sites on FPn, leading to convertase assembly. Unlike on cell surface, P* does not contribute to amplification of the pathway through additional binding sites in plasma; however, complement activation in plasma is enhanced by Properdin due to increased binding between C3bP and FB, and stabilization of C3-convertase.

The effective number of C3b binding sites on a Properdin oligomer was estimated using the weighted average of the ratios of the oligomers (dimers, trimers, and tetramers) and is approximately 3 ((22*2 + 52*3 + 28*4)/(22 + 52+28) = 3.06). The oligomer, denoted as FPn in the model, binds to surface-bound C3b, C3bBclose, C3bBopen, C3bBb, or iC3b, and generates two additional binding sites (P*) on the cell surface for convertase assembly (Figure 2). C3b and its complexes, in plasma as well as on the cell surface, can bind with P* and continue the assembly of the C3-convertase. FPn is assumed to behave in a similar manner in plasma and provides three binding sites to C3b, its complexes, and inactive cleavage products. The binding affinity of C3b to Properdin is more than the order of the magnitude higher than FB (0.0345 µM (DiScipio, 1981)) with a stable half-life of 23 min. Furthermore, Properdin binds to C3-proconvertases and convertases better than how it binds to C3b (association rate constant assumed 2.5 and 15 times that of C3b, respectively, inferred from the study by Hourcade (2006). All Properdin-bound proteins participate in the same reactions as non-Properdin proteins for binding or cleavage by FB, FD, FH, FI, and other regulators, however, with different kinetic rate constants in some reactions. Properdin-bound C3b associates five times faster with FB than nascent C3b (Hourcade, 2006) and with a reduced affinity with FH (Medicus et al., 1976). The kinetic rate constants for all subsequent interactions with P* are assumed to be the same as the 1st binding event to the oligomer FPn. Although steric hindrance might lower the binding rates for the 2^nd^ and 3^rd^ binding events, or alternatively, the cross-linking effect on C3bBb binding to multiple Properdin-binding sites may progressively increase the stabilization of the convertase (Alcorlo et al., 2013), these effects are not considered in the model.

The availability of multiple binding sites on Properdin oligomers for the assembly of the C3-convertases as well as reactions with several plasma and surface-bound proteins leads to rapid amplification, and it is a key component of complement activation.

#### 2.1.2 Terminal Pathway and its Regulators

The terminal pathway is initiated by the cleavage of C5 to C5a and C5b by the alternative pathway C5-convertase. C5a is a potent, short-lived anaphylatoxin and C5b can bind to C6 and perpetuate the formation of the MAC that causes cell lysis. However, only a portion of the newly generated C5b binds to C6 forming C5b6 before its binding site degrades. Since C5b6 is a stable complex, the model assumes the formation of this complex as irreversible. The newly generated C5b6 can bind to C7, creating the C5b-7(meta) complex that possesses a meta-stable binding site for the surface of the target cell.

The metastable binding site of C5b-7(meta) can either bind to the target membrane or become inactive in the solution. C5b-7(meta) deposition on the surface is inhibited by clusterin (Cn), vitronectin (Vn), and C8 in the plasma. The C5b-7 complexes that have lost the ability to bind to the cell surface are collectively denoted here as soluble MACs or SC5b-9 complexes. The formation of C5b-7 on the surface of a target cell represents a key point of regulation in the terminal cascade. Once bound to the target surface, C5b-7 can bind to C8 and up to 16 C9 units, forming MACs of various sizes denoted in the model as C5b-9_i_ (where 1 ≤ i ≤ 16). The binding rate of C9 molecules to C5b-8 and C5b-9_i_ is assumed to be equal based on literature evidence (Müller-Eberhard, 1988). The accumulation of MACs (C5b-9i) causes cell lysis as a function of MAC per cell surface (MAC/cell) and a cell-dependent parameter “K_m_lysis,” which denotes the number of MAC per cell that leads to 50% cell lysis.

The formation of C5b-9 complexes on the surface is inhibited by the membrane-bound regulator CD59, and it acts by inhibiting the C5b-8-catalyzed insertion of C9 into the lipid bilayer (Meri et al., 1990). CD59 is widely expressed in different cell types, including erythrocytes (Holguin et al., 1989a; Sugita et al., 1988), and is missing or reduced in PNH (Holguin et al., 1989b), causing the lysis of erythrocytes. CD59 binds tightly to the C9 binding site on C5b-8 and on C5b-9_i_, thus competing with and inhibiting the binding of subsequent C9 units. Meri et al. (1990) estimated the number of CD59 to be 25,000 per human erythrocyte.

Clusterin and vitronectin bind to C5b-7(meta) and prevent its binding to the cell surface inhibiting MAC formation and subsequent cell lysis. C5b-7(meta) bound to Cn and Vn in the fluid phase, denoted as C5b-7:Cn and C5b-7:Vn, and can continue to bind to C8 and C9 to assemble soluble MACs. In addition, Cn and Vn bind to C5b-8 and C5b-9_i_ both in the fluid and on the cell surface to prevent binding to C9 and inhibit C9 polymerization (Preissner et al., 1989; Tschopp et al., 1993; Hadders et al., 2012). The affinity of Cn for binding to C5b-8 and C5b-9_i_ has been adopted based on a previous modeling work (Korotaevskiy et al., 2009). In the absence of quantitative estimates for kinetic rate constants for vitronectin, the same values are adopted as for clusterin.

#### 2.1.3 Other Regulatory Effects

FH and FI are prominent negative regulators of the alternative pathway. In addition to binding with C3b to prevent the C3-convertase assembly, FH accelerates the decay of C3-convertases of the alternative and classical pathways (Seya et al., 1985; Bernet et al., 2004). Based on literature data (Weiler et al., 1976), the presence of FH reduces the half-life of nascent and FP-stabilized convertases by almost 4 and 2.5 times, respectively. As discussed previously, FH and FI also compete with FB for binding to C3b(meta) and lead to its cleavage into inactive C3b products such as iC3b, C3dg, and C3c.

Similar to FH, the surface receptor CR1 serves as a cofactor for the FI-mediated cleavage of C3b while also accelerating the decay of the alternative pathway convertases. Low levels of CR1 are associated with autoimmune diseases such as systemic lupus erythematosus and rheumatoid arthritis. Furthermore, the different CR1 isoforms appear to contribute to the decay acceleration and FI-mediated cleavage to a similar degree (Seya et al., 1985); therefore, different isoforms are not considered in the model.

The alternative pathway convertases are also degraded by DAF or CD55. Unlike CR1, DAF does not serve as a cofactor for FI-mediated cleavage. The effect of DAF on the decay of C3-convertase has been included based on the literature studies (Harris et al., 2005) on both cell surface and plasma in the model.

#### 2.1.4 Baseline Complement Model Simulation

The baseline simulation of the complement model represents a human *in vivo* “healthy state” without any disease pathologies and captures the healthy levels of major complement proteins within the known ranges. The levels of complement proteins and their serum half-lives are summarized in Table 1. For complement proteins where data on half-life were not available, a 2-day half-life has been assumed based on the known ranges of the half-lives for other complement proteins. The synthesis rates of proteins were estimated to maintain the healthy levels in baseline simulations.

**TABLE 1:** Complement protein levels in healthy state in humans and their half-lives.

Protein	MW	Serum concentration^a^(micromole/L)			Half-life	Half-life references
	(kDa)				(hour)	
		Min	Max	Average	*Unless specified*	
C3	185	5.405	8.108	6.486	49–69	Sliwinski and Zvaifler (1972)
C5	190	0.289	0.595	0.395	34.65	Alper and Rosen (1984)
C6	105	0.514	0.686	0.610	30–50 min	Schaller et al. (2008)
C7	92.4	0.530	0.758	0.606	61	Schaller et al. (2008)
C8	151	0.331	0.530	0.364	48	Assumed
C9	71	0.662	0.986	0.845	48	Assumed
FB	93	1.828	2.774	2.151	34.65	Alper and Rosen (1984)
FD	24	0.042	0.083	0.058	0.87	Pascual et al. (1988)
FH	155	1.613	3.639	3.226	6 days	Licht et al. (2005)
FI	88	0.386	0.386	0.386	45	Møller Rasmussen et al. (1988)
FP^b^	53	0.094	0.283	0.094	73.2	Alper and Rosen (1984)
Vn	70	2.857	5.714	6.786	48	Assumed
Cn	80	3.125	5.250	3.750	48	Assumed

a “Human Complement Proteins.” https://www.complementtech.com/catalog/human-complement- proteins/ (accessed Jun. 15, 2017).

b FP, trimer concentration (FPn) = 0.094/3 = 0.031 μmol/L.

### 2.2 Methods for *In Vitro* Complement Assays

#### 2.2.1 Alternative Pathway Assays

The generation of C3a, Ba, and iC3b, in the presence and absence of FP, was measured in an *in vitro* assay using purified alternative pathway components (all purchased from Complement Technologies, Tyler, Texas, United States). C3 (final 300 μg/ml), C3b (final 5 μg/ml), factor B (final 50 μg/ml), factor D (final 350 ng/ml), factor H (final 125 μg/ml), and factor I (final 8.5 μg/ml) were mixed in an assay buffer (PBS/14 mM MgCl_2_) in the presence or absence of Properdin (1.25 μg/ml) and placed in a 37°C water bath. Samples were removed at each time point (5, 10, 15, 30, and 45min; 1, 1.5, 2, and 3 h) and placed in a fresh tube containing 2 μl of 10x stopping solution (100 mM EDTA/100 μM GSK3528001A, a small-molecule factor B inhibitor) and placed on wet ice. The samples were diluted 1/5 with PBS and stored at −80°C until cleavage fragment analysis. C3a was measured using the Complement C3a Human ELISA Kit (Invitrogen, BMS 2089, lot: 123684009), and the samples were further diluted 1/75 in the kit sample diluent. Ba was measured using the MicroVue Ba fragment EIA (Quidel, A034, lot: 064580) and the samples were further diluted 1/500 in the kit sample diluent. A custom sandwich ELISA (method in Supplementary Material) was developed to measure iC3b using neo-epitope specific monoclonal antibodies (capture: antihuman C3 clone bH6; detection: antihuman C3dg/iC3b/C3g clone 9; both Hycult).

All complement proteins (human biological samples) for this and other assays were sourced ethically, and their research use was in accordance with the terms of the informed consents under an IRB/EC approved protocol.

##### 2.2.2. Terminal pathway assays

The titration of purified C5b6, C7, C8, and C9 was performed in a reactive lysis system using guinea pig erythrocytes. For each assay plate (C7, C8, and C9 titrations), 2 ml of guinea pig erythrocytes (supplied in Alsever’s solution, TCS Biosciences) were washed in PBS and pelleted (500xg, 5 min). After aspiration of the supernatant, 120 μl of the cell pellet were suspended in 6 ml C5b6 in PBS (1 μg/ml) and incubated for 5 min at 37°C in a water bath. For the C8 and C9 titrations, C7 (1 μg/ml) was added directly to the C5b6-coated erythrocytes to generate C5b7-coated erythrocytes and incubated for 15 min at 37°C, followed by two washes with PBS. C8 (or C9) was serial-diluted in wells of a round-bottom polypropylene plate (50 μl per well), and C9 (or C8) was diluted to 3 μg/ml (or 1 μg/ml) and 50 μl added to the relevant wells in the serial-dilution plates for C8 (or C9). 50 μl of C5b-7-coated erythrocytes were then added to each well and the reactions incubated on a plate shaker for 30 min at 37 °C. For the C7 titration, C7 was diluted to 10 μg/ml, serial-diluted in the wells of a round-bottom polypropylene plate and 200 μl of suspended C5b6-coated erythrocytes added to each well, and the plates were covered and incubated for 15 min at 37°C, followed by two washes in PBS and the resuspension of the pellets in 150 μl PBS. 50μl of the cells were then transferred to a fresh plate containing 100 μl of C8 and C9 (at 1 and 3 μg/ml, respectively), and the reactions were incubated on a plate shaker for 30 min at 37°C. Cells mixed with 100 μl H_2_O/0.01% Triton-x-100 (Sigma-Aldrich) or 100 μl PBS only were used as 100% lysis and background lysis control, respectively. After incubation, the plates were centrifuged for 5 min at 1000xg, the 100 μl supernatant was transferred to a microtiter plate, and the absorbance was read at 540 nm. For analysis, the background lysis was subtracted from all samples, and the percentage lysis relative to the 100% lysis control was calculated.

### 2.3 Validation of the Complement Model Simulations

**FIGURE 3:**
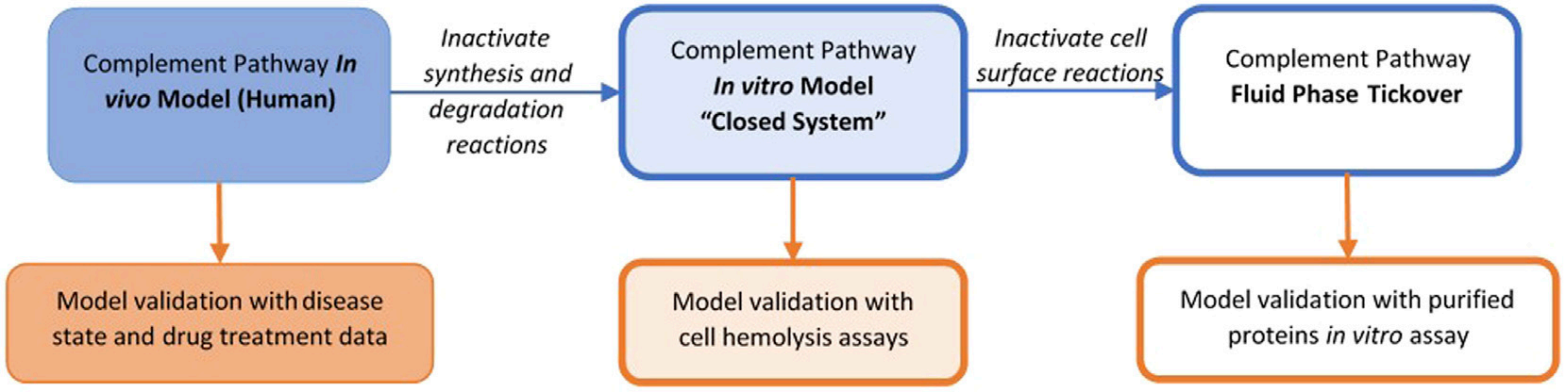
Representations of the complement model for validation with different datasets.

The complement model dynamics were validated using *in vitro* assay data for the alternative and terminal pathways as well as patient disease state data. A “unit testing” approach was used for validating the QSP model dynamics as model sub-components were compared with different datasets separately. A schematic for model simulations for *in vitro* and *in vivo* data is shown in Figure 3. The *in vitro* assays can be conducted using purified complement proteins with or without target cells where the activation of the pathway is measured using proteolytic cleavage products such as C3a, Ba, iC3b, and cell hemolysis. To simulate the *in vitro* assays, the synthesis and degradation reactions in the MATLAB SimBiology model were set as “inactive” to create a “closed system.” If the cells were not used in the assay, all the reactions for cell surface variables were also set as “inactive,” resulting in a simplified “fluid phase tickover” model. The initial conditions in the model were set as the concentrations of the purified proteins or cells used in the *in vitro* assays, and the rest of the state variables were set to 0. After any washing step in the assays with cells, the fluid compartment concentrations are set to 0, while the cell surface concentrations are unaffected before the next protocol step is simulated. The model was simulated for the assay protocols, and the time-course of cleavage fragments or cell hemolysis was compared against experimental data.

The overall validation of the model dynamics has also been performed against data for disease pathologies and drug treatments for completeness. Thus, varied representations of the same complement pathway model, for example, fluid-phase model, closed system, or human *in vivo* system, supported model validation across several data types with parameter values kept the same across all the representations.

## 3 Mathematical Modeling Results

### 3.1 Model Validation With *In Vitro* Data

#### 3.1.1 Alternative Pathway Assays

**FIGURE 4:**
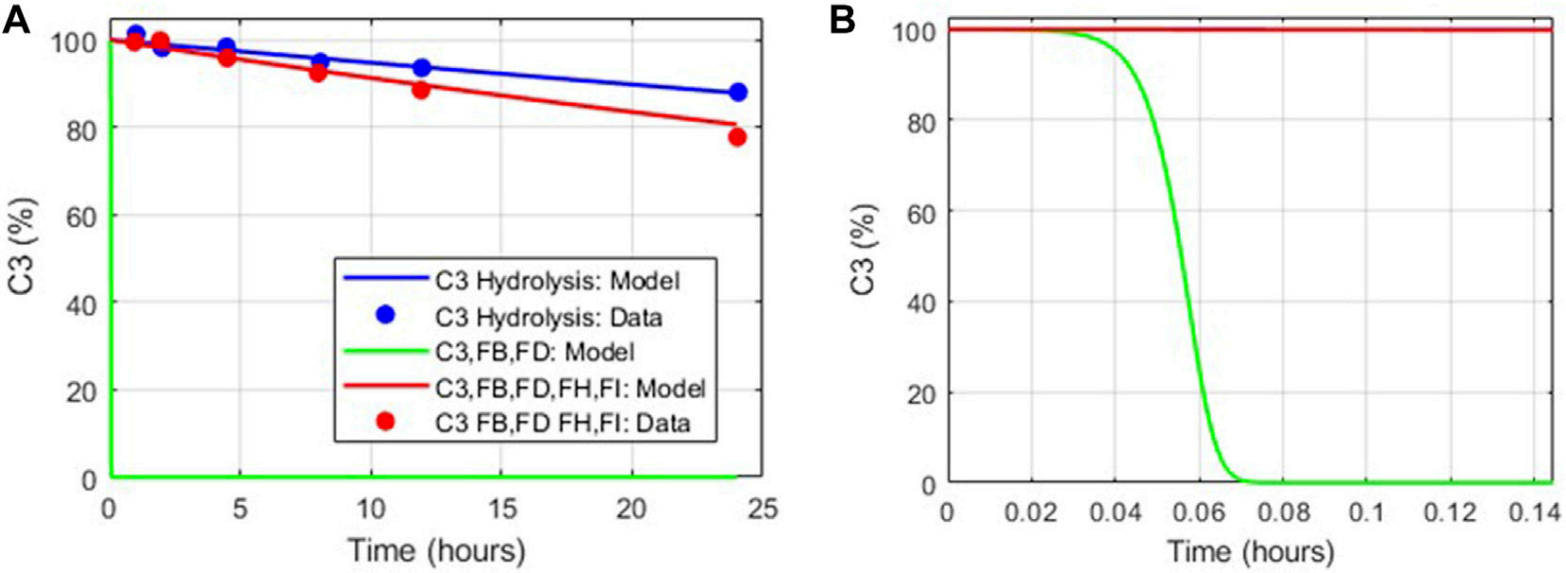
Comparison of the tickover complement model dynamics with *in vitro* data (Pangburn et al., 1981). **(A)** Model simulations match *in vitro* data for C3 hydrolysis alone (blue), with FB, FD added (green) and with FH, FI, FB, and FD added (red). **(B)** Zoomed in view of model simulations for decay in C3 hemolytic activity in the presence of FB, FD (green).

The dynamics of tickover reactions in the complement model was assessed based on the literature data (Pangburn et al., 1981) for the decay of C3 hemolytic activity due to spontaneous hydrolysis alone, with purified proteins FB and FD, and with FB, FD, FH, and FI. Aligned with the literature data, the hemolytic activity of C3 due to spontaneous hydrolysis was predicted to decline at a slow rate (Figure 4A), and in the presence of FB and FD, all C3 hemolytic activity was lost within minutes (Figure 4B). However, the regulatory activity of FH and FI in addition to FB and FD led to the controlled decay of C3 hemolytic activity, but it was faster than that due to hydrolysis alone. The value of the parameter for only the forward binding rate of C3b to FB was adjusted within the literature reported values (1.74–4.74 L/(micromole*minute)) to match the experimental data.

**FIGURE 5:**
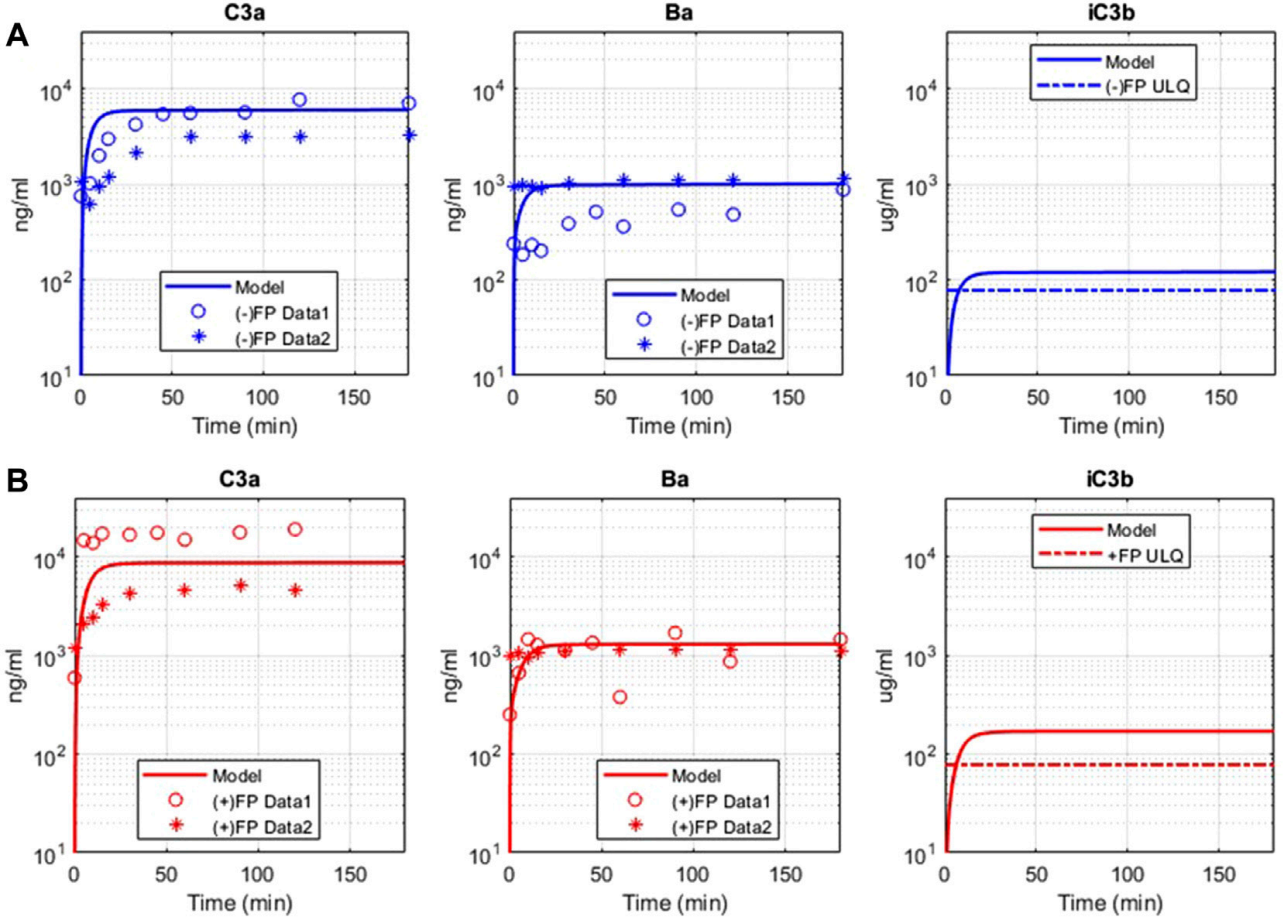
Comparison of model simulations with GSK *in vitro* assay data for alternative pathway. Model results are denoted by solid line, and the assay results are from two sets of independent, identical experiments, denoted as “Data 1” (o) and “Data 2” (*). **(A)** Model simulation and *in vitro* data for alternative pathway assay without Properdin (−) FP for C3a, Ba, iC3b. **(B)** Model simulation and *in vitro* data for alternative pathway assay with Properdin (+)FP for C3a, Ba, and iC3b.

The complement pathway fluid-phase tickover model was also compared with GSK *in vitro* data for the pathway activation *via* the formation of cleavage products C3a, Ba, and iC3b. There is a significant increase in C3a, Ba, and iC3b due to complement activation in the assay, and the model predictions match with the observed data for C3a and Ba for both the experimental conditions with and without FP (Figure 5). The model predicted that the levels of iC3b will be above the ULQ which was also observed in the assay. The value of the parameter for only the forward binding rate of C3b to FH was adjusted within the values from the literature (6.08–66.48 L/(micromole*minute)) to match the experimental data. The mechanistic model captures the increased pathway activation in the presence of FP (Figure 5B) without any further changes in the parameter values or mechanistic implementation of FP dynamics.

Please note that the model parameters were not fitted against the *in vitro* data. The model was able to describe the *in vitro* datasets by adjusting the parameter values within their known ranges, thus validating the model implementation of the complement processes and literature derived kinetic parameters. Moreover, there is also variability observed between the experimental datasets. The *in vitro* AP assays are rapid reactions, and there are several factors that can cause variability. These factors include small differences in the manual addition of reactants, addition of stop solution, temperature, thawing of proteins, and the presence of small amounts of aggregate. The experiments included were also performed on different days.

#### 3.1.2 Terminal Pathway Assays

**FIGURE 6:**
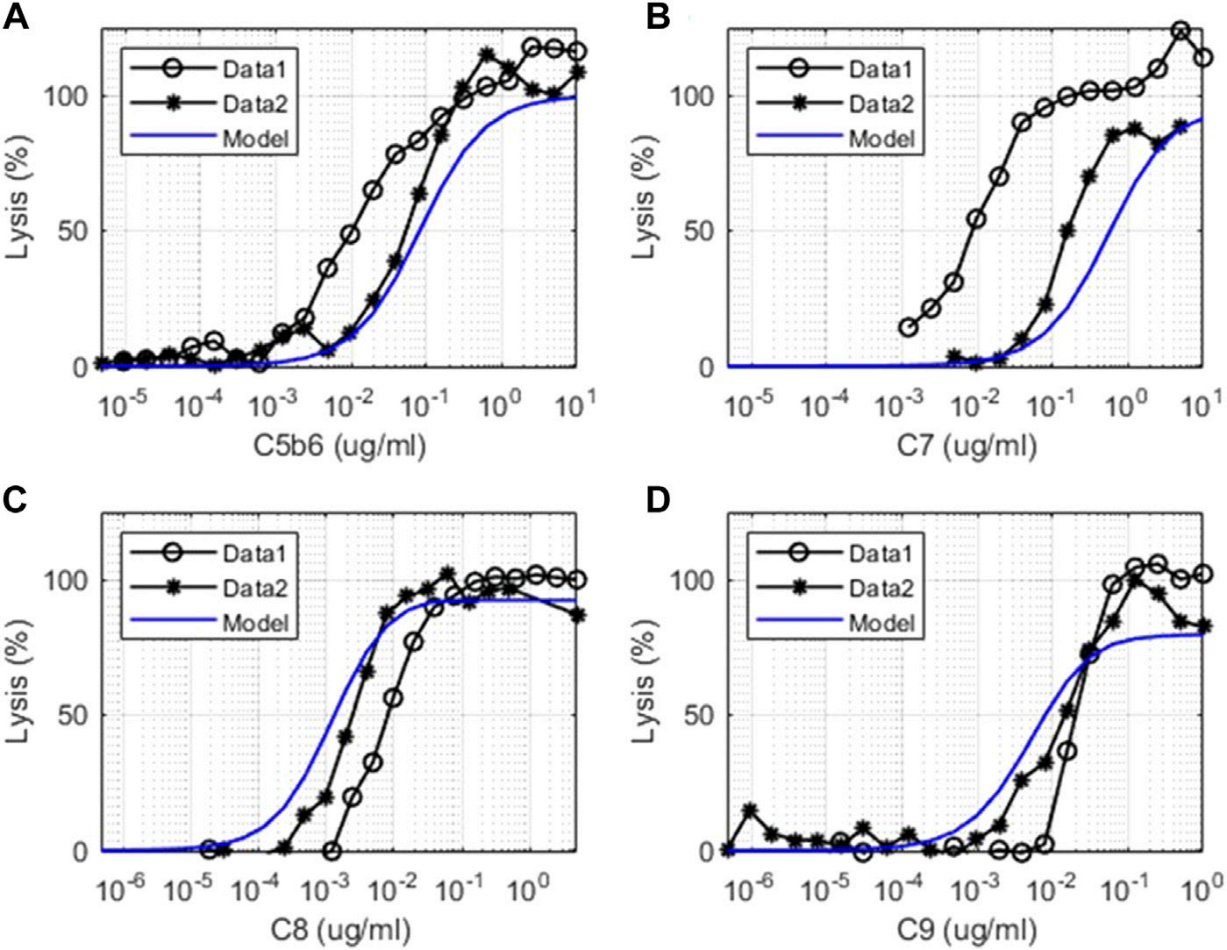
Comparison of model simulations with *in vitro* assay data for terminal pathways. Model simulation results are denoted by solid blue line, and the terminal pathway assay results are from two sets of independent, identical experiments denoted as “Data 1” (o) and “Data 2” (*). Dose–response of cell lysis is shown for different concentrations of **(A)** C5b6 (μg/ml), **(B)** C7 (μg/ml), **(C)** C8 (μg/ml), and **(D)** C9 (μg/ml).

There is increased lysis of guinea pig erythrocytes (GPE) observed in terminal pathway assays due to the formation of the MAC on the cell surface by the action of terminal pathway proteins derived from human plasma. The *in vitro* complement model captures the level of hemolysis observed in GPE due to the titration of the levels of C5b6, C7, C8, and C9 complement proteins in terminal pathway assays (Figure 6). The cell-dependent parameter “K_m_lysis” was determined to be 0.5 for GPE based on the C5b6 hemolysis assay and kept the same for all other terminal pathway titrations. These assays provide a comprehensive validation of the parameters in the terminal pathway of the model.

The experimental system used for the *in vitro* terminal pathway lysis is a rapid lysis system using GPE. The guinea pig cells are used instead of human erythrocytes as they are void of cell surface regulators that would control human terminal pathway proteins. Healthy human erythrocytes would not lyse effectively. As the experimental system is very sensitive, variation between experiments can be derived from cell batches, small differences in cell number, or time of the cells in storage. The experiments shown were also performed on different days.

### 3.2 Model Simulations for Humans

In addition to unit testing of the complement pathways with *in vitro* data, the overall model simulations were also compared against human *in vivo* data for the validation of the model dynamics.

#### 3.2.1 Model Validation With Patient Disease States

Deficiency or mutations in regulators of the complement pathway have been implicated in several diseases. Cell surface regulators (CR1, DAF/CD55, and CD59) and FH in plasma were reduced in the complement model to assess the effects on pathway activation *via* an increase in complement fragments, soluble and surface MACs, and lysis of cells. The cell surface regulators were reduced by 99%, representing almost the complete loss of function, and FH was reduced by 50% of their baseline values to simulate their deficiencies or mutations observed in autoimmune diseases (Iida et al., 1982; Rougier et al., 1998; Brodsky, 2014).

**FIGURE 7:**
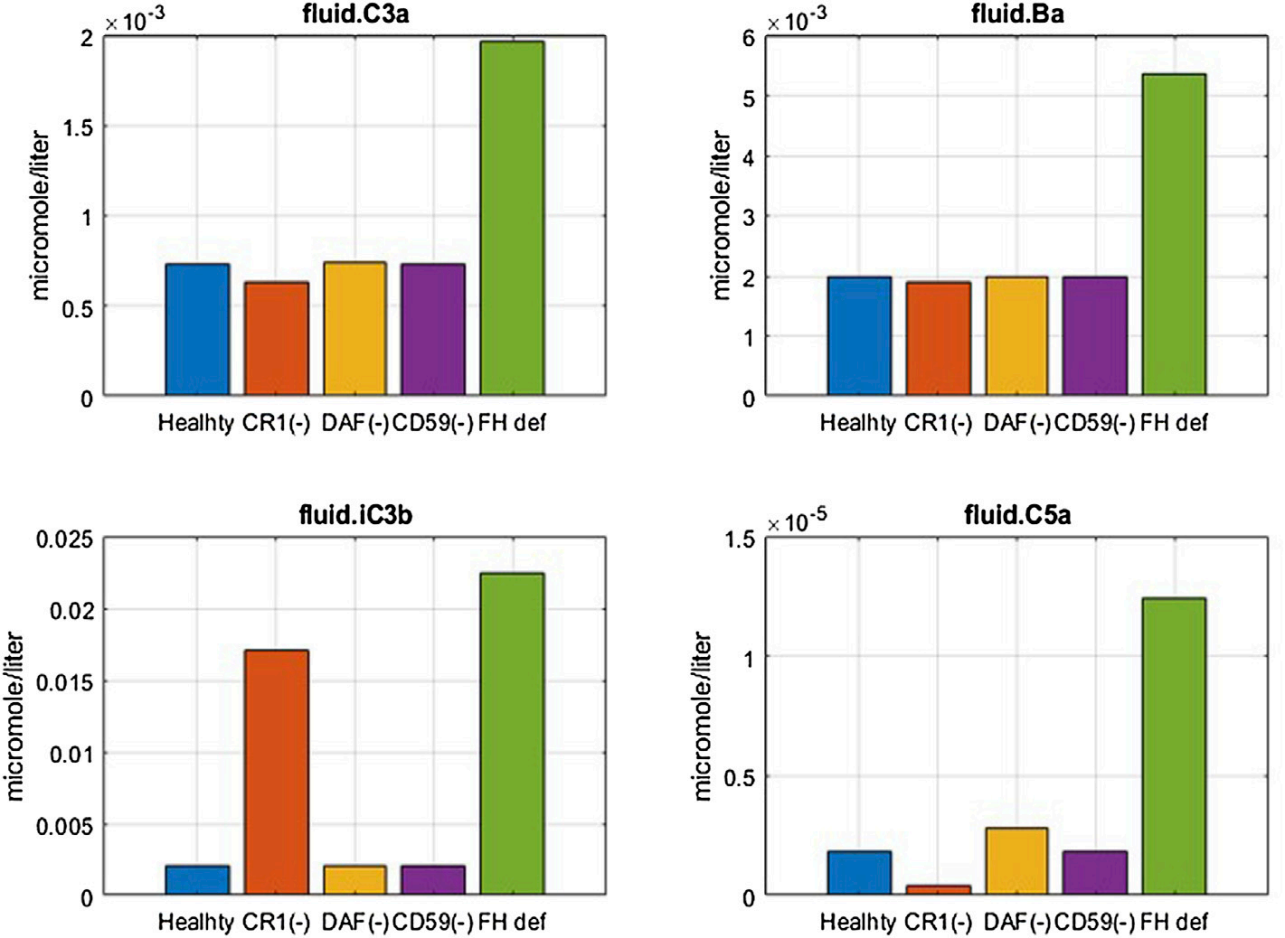
Effect of 99% reduction in cell surface regulators, CR1, DAF, and CD59, and 50% reduction in fluid phase regulator FH on complement fragments C3a, Ba, iC3b, and C5a.

**FIGURE 8:**
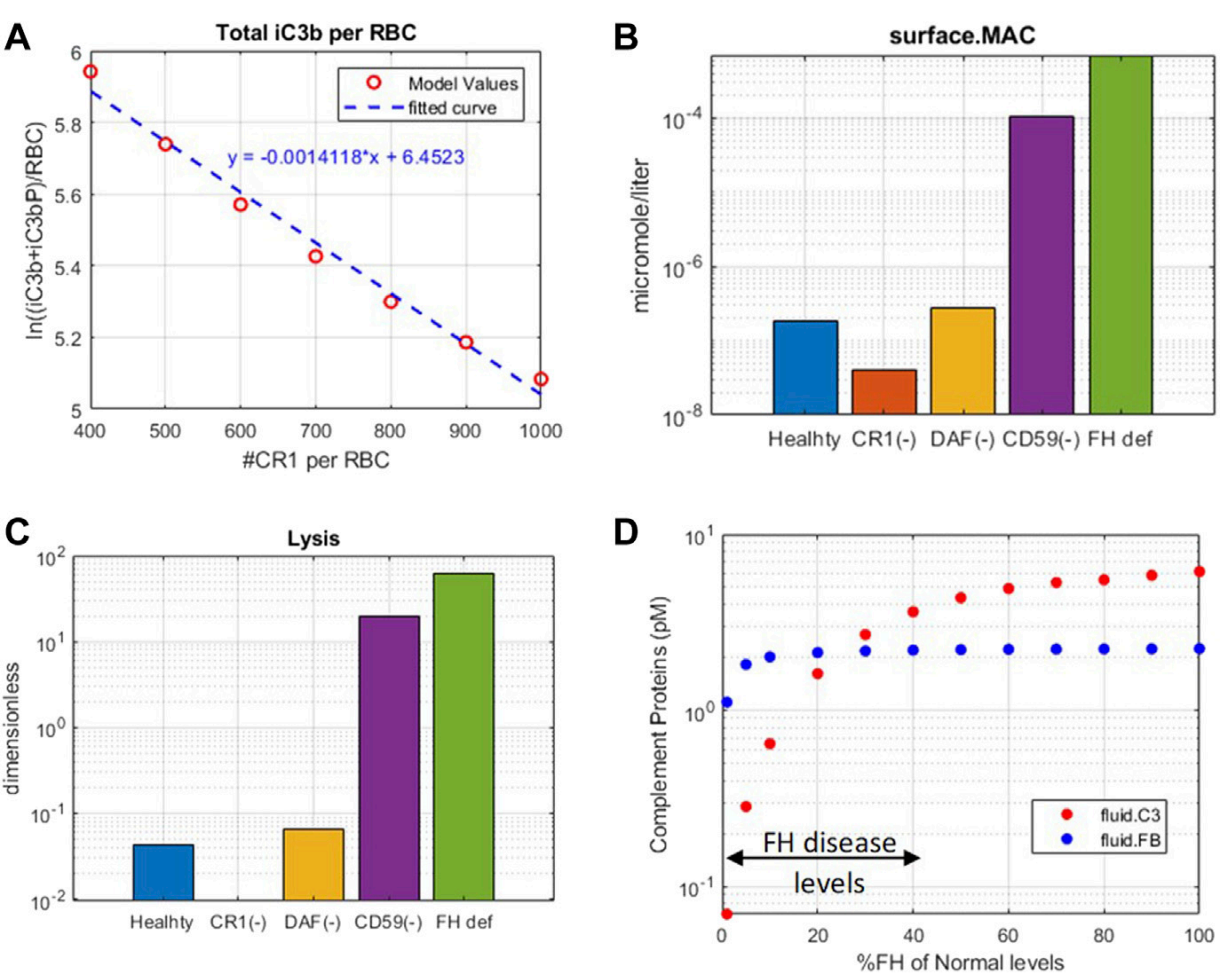
Complement model predictions for **(A)** correlation between iC3b + iC3bP deposition on erythrocytes and levels of CR1, **(B)** surface MAC, **(C)** lysis of host erythrocytes due to 99% reduction in CR1, DAF, and CD59 and 50% reduction in fluid-phase regulator FH, and **(D)** consumption of complement proteins C3 and FB due to FH deficiency ranging from 0 to 99%.

Model simulations show that reduction in CR1 leads to a significant increase in the surface and plasma levels of iC3b (Figure 7) because it acts as a cofactor for factor I-mediated cleavage of C3b and C3-convertases. An increase in iC3b and other C3 fragments may contribute to complement-driven immune activation in diseases such as systemic lupus erythematosus (SLE) where patients have reduced levels of CR1 in erythrocytes (Iida et al., 1982). In fact, the model also predicts a negative linear correlation between the levels of CR1 receptors with deposition of C3 fragments on erythrocytes (Figure 8A) similar to the relationship reported in the literature for SLE patients (Ross et al., 1985).

Reduction in CD59 leads to two to three orders of magnitude increase in surface MACs (Figure 8B) and soluble MACs (data not shown), causing significant lysis of erythrocytes (∼20%) (Figure 8C). These simulations are aligned with increased lysis of erythrocytes observed in PNH disease where erythrocytes are highly vulnerable to complement-mediated lysis due to a reduction, or absence, of CD59 and DAF/CD55. However, model simulations show that reduction in DAF does not cause an increase in the MAC or lysis (Figure 8B,C) but may contribute to inflammation through some increase in complement fragments such as C5a (Figure 7). In line with these modeling results, it has been reported that the reduction in CD59 contributes more to PNH pathogenesis than DAF as there are patients with DAF deficiency but normal CD59 expression who do not have clinically evident hemolytic disease (Wilcox et al., 1991; DeZern and Brodsky, 2015).

A reduction in FH causes a significant increase in several complement fragments, MACs, and cell lysis (Figure 7, Figure 8). Modeling results also show a reduction in other complement proteins such as C3 and FB due to an increased consumption from the persistent activation of the alternative pathway (Figure 8D). The consumption of complement proteins increases significantly at higher FH deficiency levels. These results are consistent with complement protein deficiencies observed in diseases linked to FH mutations such as aHUS, glomerulopathies, and acute infections (C3: 5–68% and FB: 35–100% of normal (Nielsen et al., 1989; Vogt et al., 1995; Rougier et al., 1998)), where FH levels or function vary from 0 to 40% of normal levels.

It is interesting to note that CD59 and FH deficiencies have the strongest effects on cell lysis, which is seen in PNH and aHUS patients where CD59 deficiency and FH deficiency, respectively, have been indicated. FH deficiency also causes an increase in complement fragments (C3a, Ba, iC3b, C5a, *etc*.) which drive inflammatory processes such as immune cell attraction, opsonization, and phagocytosis of host cells, leading to worsening of autoimmune diseases in addition to lysis of host cells.

#### 3.2.2 Effect of Complement Targets on Disease States

The effect of potential drug treatments for reducing disease severity driven by complement pathway activation was evaluated using the computational model. CD59 and FH reduction leads to significant amount of cell lysis, and increase in complement fragments and MAC proteins. Complement model simulations for CD59 and FH reduction represent a semi-mechanistic way of simulating PNH and aHUS disease pathologies, respectively. QSP model simulations were used for assessing the effect of potential treatments to reduce the complement activation for these pathologies by reducing the levels of key complement proteins and fragments to 10 and 1% of the normal levels, that is, 90% and 99% inhibition, respectively, from the baseline, which are the typical levels of target inhibition aimed during drug development. CD59 reduction was assumed to be at 99% of its baseline value, and FH reduction at 40% of the baseline aligned with a reduction in function or levels observed in diseases (Rougier et al., 1998; Brodsky, 2014).

**FIGURE 9:**
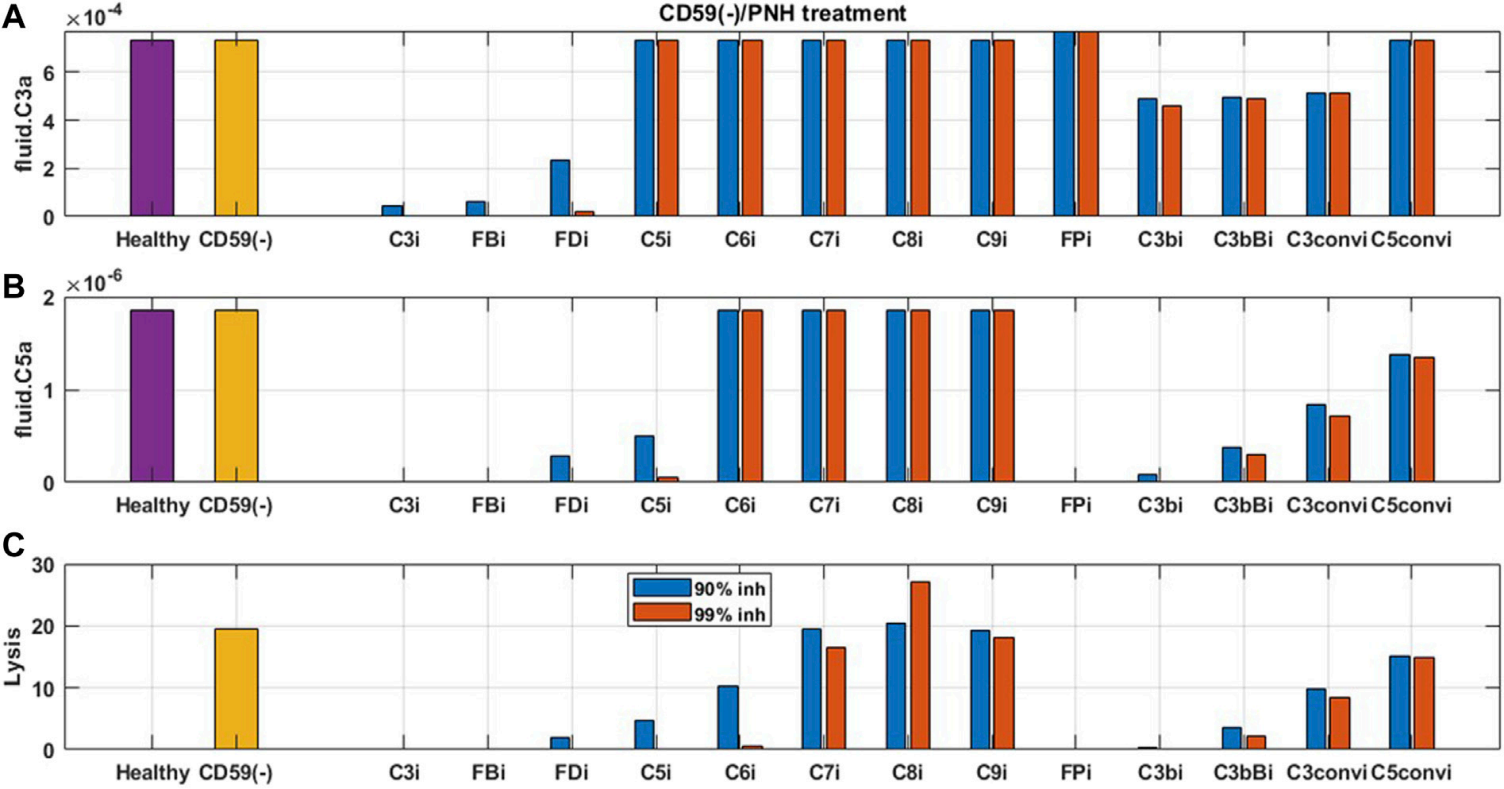
Model predictions for the effect of 90 and 99% inhibition of complement proteins (C3, FB, FD, C5, C6, C7, C8, C9, FP, C3b, C3bB, C3-convertase (C3conv), and C5-convertase (C5conv)) on complement activation driven by CD59 deficiency or knockout such as in PNH disease.

For complement activation driven by CD59 reduction, significant lysis of erythrocytes was observed, but there is no increase in upstream biomarkers such as C3a and C5a (Figure 9). For reducing this activation, 90% inhibition of C3, FB, FD, C5, C6, FP, C3b, C3Bb, and C3-convertase (C3bBb and C3bBbP) leads to a significant reduction in cell lysis (Figure 9C). Moreover, C6 levels needed to be reduced by 99% for complete reduction in cell lysis which may be infeasible with real-world drug compounds. Modulation of alternative pathway proteins C3, FB, FD, C3b, C3bB, and C3-convertase also reduces the complement fragments C3a (Figure 9A), iC3b, and Ba (data not shown); however, it may not be important for PNH disease as the levels of these inflammatory mediators were not predicted to be elevated in comparison to healthy control.

**FIGURE 10:**
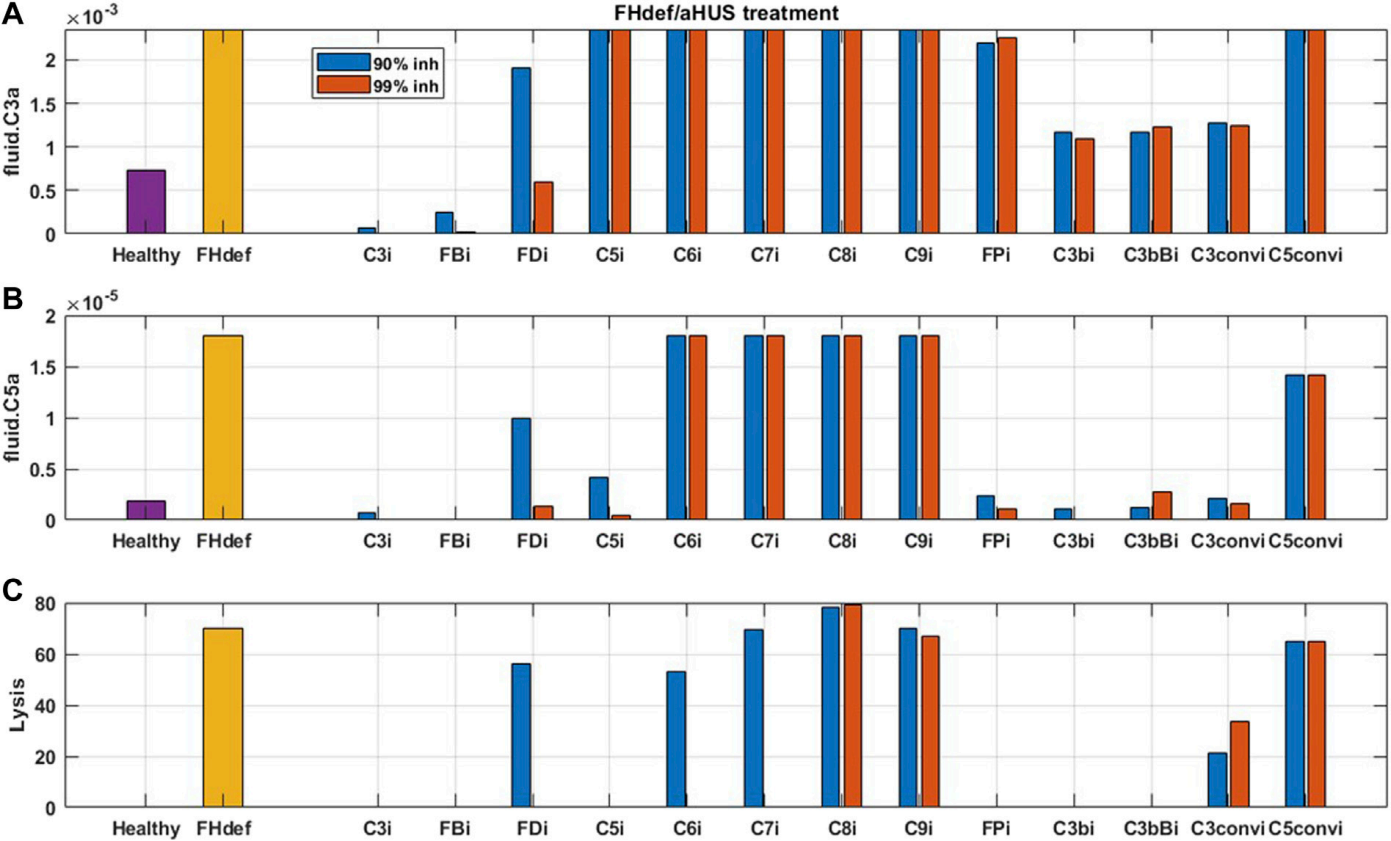
Model predictions for the effect of 90 and 99% inhibition of complement proteins (C3, FB, FD, C5, C6, C7, C8, C9, FP, C3b, C3bB, C3-convertase (C3conv), and C5-convertase (C5conv)) on complement activation driven by 40% FH deficiency such as in aHUS.

For reducing complement activation due to FH deficiency, drug compounds targeting C3, FB, C5, FP, C3b, C3bB, and C3 convertase are predicted to be efficacious in reducing disease severity (Figure 10). While 99% inhibition of FD, C6, and C7 is also predicted to reduce cell lysis, it may be infeasible from a drug-development perspective due to high drug dosing requirements. In addition, C6 and C7 inhibition does not lead to a reduction in alternative pathway fragments and may provide only partial efficacy in reducing complement-driven immune activation in aHUS.

As expected, reduction in terminal pathway proteins C6, C7, C8, and C9 does not lead to a reduction in alternative pathway biomarkers such as C3a, C5a (Figure 9, Figure 10), iC3b, and Ba (data not shown). It is also interesting to note that the inhibition of alternative pathway cleavage fragments C3b, C3bB, and C3-convertase has a stronger effect on C5a and cell lysis than that of C3a in plasma. This is because reduction in these alternative pathway fragments shifts the contribution of C3 cleavage from C3-convertase C3bBb to the fluid tickover convertase C3(H_2_O)Bb, thus still maintaining the production of C3a in plasma but causing a reduction in C5a which is formed by the cleavage of C5 by C5-convertase on the cell surface downstream of surface C3-convertase.

#### 3.2.3 Dosing Tractability of Complement Targets

The simulations in Sections 3.2.1 and 3.2.2 assume sustained inhibition of targets for a prolonged duration. These assessments are invaluable for providing a preliminary assessment for target validation efforts in drug discovery. However, the sustained high level of target inhibition cannot be attained with real-world drugs because of factors such as rapid metabolism of the drugs *in vivo*, limits on drug doses due to toxicity effects, and inconvenience of frequent IV/SC dosing. We have evaluated the feasibility of developing drug modalities targeting specific complement proteins by assessing the dose levels and affinities needed for small molecules (SMs) and large-molecule antibody (LM-Ab) compounds to maintain reduction in free complement target levels and pathway activation. The PK parameters and affinities are assumed the same as a GSK’s tool FB inhibitor compound for the SM modality and for a typical “good” LM-Ab compound based on published PK parameters for known mAbs (Zhao et al., 2012; Ovacik and Lin, 2018) (Table 2). Note that additional modalities such as peptides, other Ab fragments, and pro-drugs were not considered for simplicity but can also be evaluated using the respective PK parameters and dose levels.

**TABLE 2:** PK model and parameters for small molecules and large-molecule antibodies.

	Unit	Small molecule	Large molecule-ab
PK model type		2-compartment	1-compartment
Absorption rate (Ka)	1/hr	3.0	5.0
Elimination rate (K10)	1/hr	0.11 (half-life: 6.3 h)	1.03e-3(half-life: 28 days)
Distribution parameter K12	1/hr	0.53	
Distribution parameter K21	1/hr	0.39	
Volume of distribution (Vd)	L/kg or L	0.54 L/kg	5 L
Bioavailability (F)		0.95	1.0
Affinity (K_D_)	ΜM	1e-3	1e-6
Drug kon	1/μM/min	6e+3	60
Molecular weight	Da	390 Da	150,000
Body weight	kg	70	70
Feasible dose range	mg or mg/kg	<= 100 mg	<=20 mg/kg
Dosing frequency		Once daily	Biweekly (two weeks)

**FIGURE 11:**
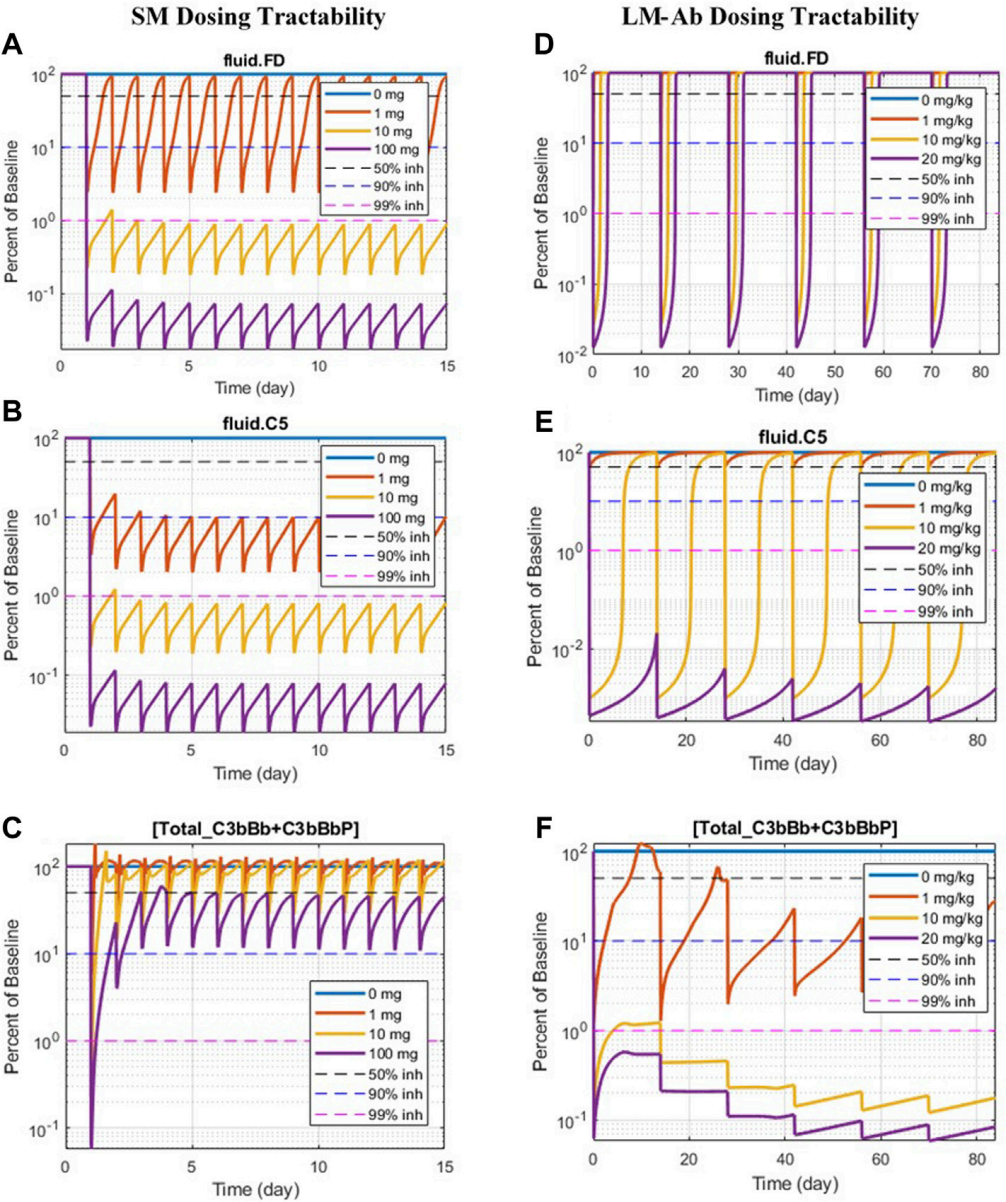
Doses of SM/LM-Ab compounds needed for engaging complement proteins. Free complement protein levels are shown for different doses of SM (0, 1, 10, and 100 mg) and LM-Ab (0, 1, 10, and 20 mg/kg). Dashed lines: 50% inhibition (black), 90% inhibition (blue), 99% inhibition (pink) from baseline. Solid lines: % free target from the baseline at different dose levels. **(A**,**D)** Blocking of FD; **(B**,**C)** blocking of C5; **(C**,**F)** blocking of C3-convertases (C3bBb + C3bBbP).

**FIGURE 12:**
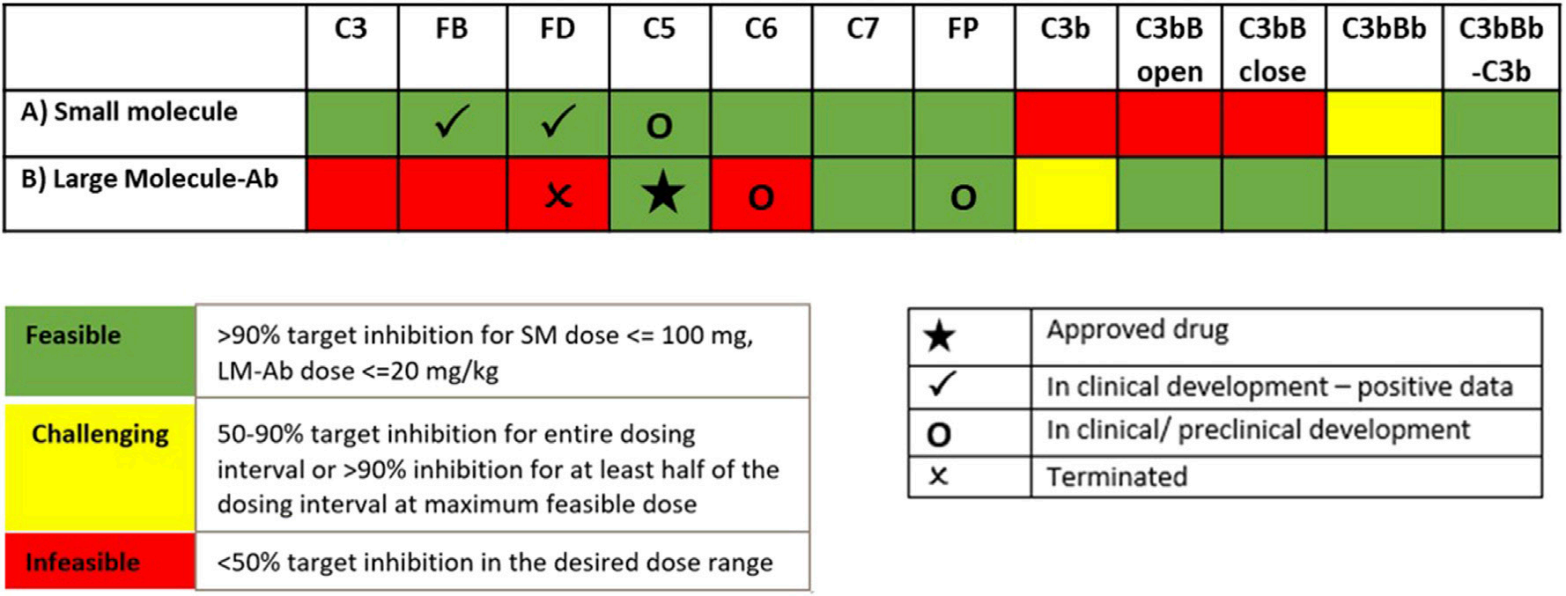
Model predictions for dosing tractability of complement proteins for SM and LM-Ab drugs.

The information on drugs in development (Mastellos et al., 2019; Zelek et al., 2019) is overlaid on dosing tractability predictions performed using the computational model (Figure 12). Several complement proteins that are present in very high concentrations or have high synthesis/turnover rates (e.g., C3, FB, FD, and C6) are challenging to block in plasma with LM-Ab compounds (Figure 12B) as the drug doses in molar amounts needed for target engagement would be higher than the feasible dose ranges (≤20 mg/kg). This also leads to faster drug clearance due to target-mediated drug disposition (TMDD) for LM-Abs. The inability to engage FD for the entire dosing interval of two weeks even with doses of 20 mg/kg of an antibody is shown in Figure 11D. Note that the feasible subcutaneous doses of LM-Ab drugs are even lower, usually <5 mg/kg, and may further restrict the target inhibition that can be attained for complement targets. On the other hand, since high molar amounts of SMs for target engagement can be administered with relatively low dose levels in milligrams due to low molecular weights, SM modalities can be used to block high-concentration complement proteins with low doses. This is illustrated using the compliment model for FD and C5 where ≥ 99% inhibition is attained for an SM dose of only 10 mg (Figures 11A, B).

The complement proteins that have a short half-life (e.g., C3b, C3bB, and C3bBb) were predicted to be challenging or infeasible to engage with SM compounds (Figure 12A). These short-lived proteins are quickly consumed or degraded in the complement pathway and thus require higher drug affinities for engagement within their short life span and for competing with other proteins in the pathway. Since affinities of SMs are lower than those of LM-Ab compounds, it is challenging to attain higher target engagement for complement fragment targets with SMs as shown for C3-convertase (Figure 11C) where only 50% engagement is attained even at the highest SM dose of 100 mg. Additionally, since the plasma levels of C3-convertase are lower, it makes the convertase an ideal target for LM-Ab modality (Figure 11F).

Several complement proteins (e.g., C5, C7, Properdin, and C5-convertases) are predicted to have dosing tractability by both small and large molecules with appropriate ranges for target concentrations, turnover, and half-lives. C5 target has the only approved antibody drug, eculizumab, as well as SM assets in development, indicating the tractability by SM/LM-Ab modalities as also predicted by the complement pathway model (Figures 11B,E). Although the approved doses needed for the LM-Ab drug eculizumab are high for PNH and aHUS indications, they still lie within the “feasible” ranges assumed in this study.

#### 3.2.4 Drug Affinities Needed for Target Engagement

A nominal drug affinity has been assumed for SMs and LM-Ab compounds for dose assessments in Section 3.2.3 at 1 nM and 1p.m., respectively. These values are at the upper limit of the feasible affinities for these modalities. Lower drug affinities may be compensated by administering higher doses of drug candidates to attain the required level of target engagement. However, since there are limits on the highest dose range for each modality, lower drug potencies can only be compensated to a certain extent. Thus, the QSP model was used to predict the minimum drug affinity that can still lead to 90% target engagement at the highest feasible dose for SM/LM-Ab compounds. This provides an estimate of the drug affinity that can be aimed during lead optimization of compounds to attain high levels of target engagement for the drug modalities.

**FIGURE 13:**
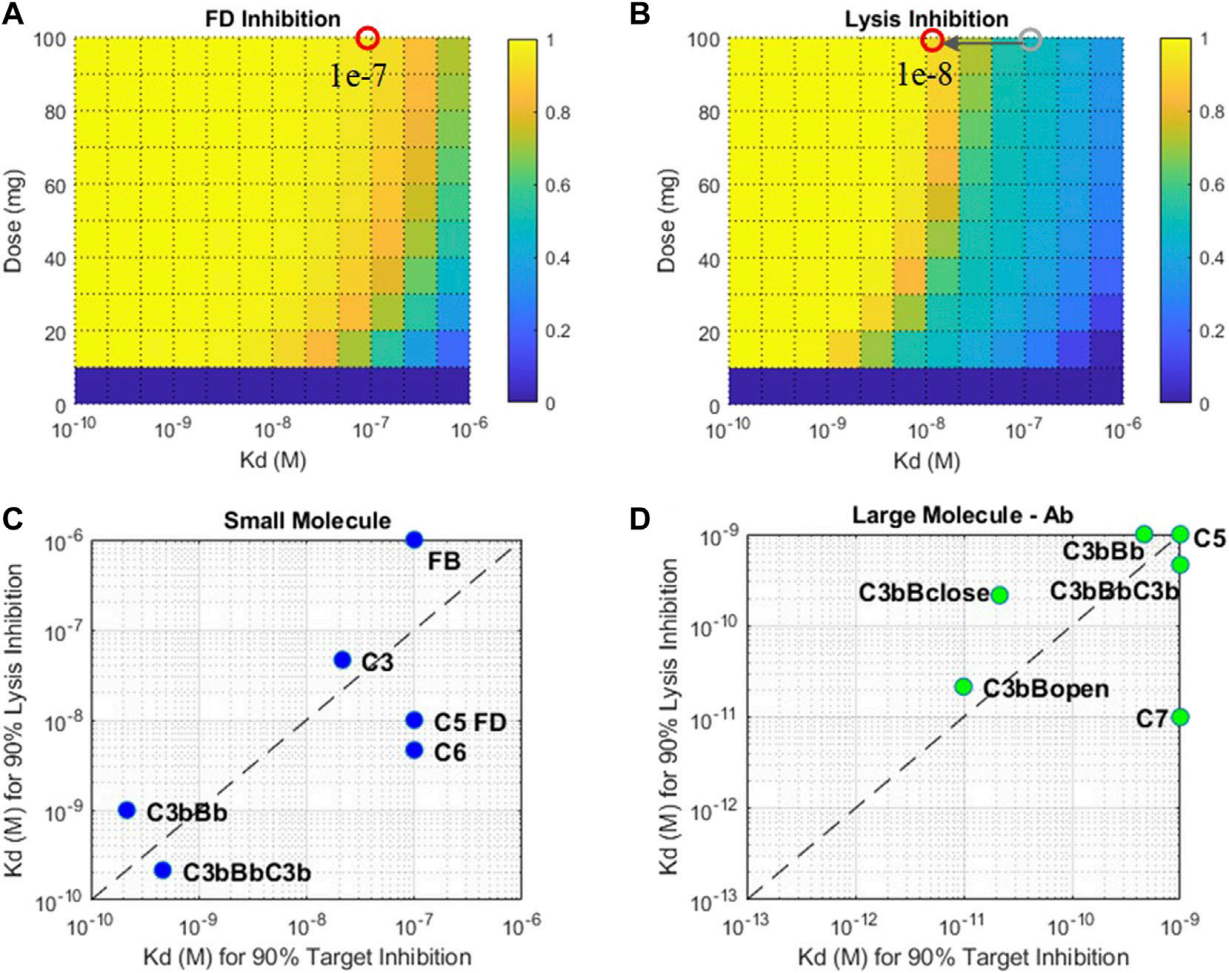
Fractional inhibition in **(A)** FD and **(B)** cell lysis at different affinities (K_D_) and doses of a SM modality. Drug affinities required for 90% target inhibition versus 90% cell lysis inhibition for **(C)** small-molecule and **(D)** large-molecule Ab modalities. Plots **(C)** and **(D)**: only the select targets that attain 90% TE and cell lysis are shown. The affinity ranges tested for SM modality—1 μM-0.1 nM and LM-Ab modality 1nM-0.1p.m. Affinity requirements predicted to be on the edges of the plots may be lower than the ranges tested.

The drug affinity requirements for 90% target engagement (TE) may be different from the affinity required for 90% reduction in the disease severity because of the pathway dynamics. This is because the level of TE required for reduction in disease severity may be much higher or lower than the customary 90% TE assumption. Thus, affinities needed for a 90% reduction in cell lysis have been estimated separately. For example, the SM affinity required for 90% inhibition of FD at the highest feasible dose of 100 mg is ∼0.1 μM (Figure 13A). But the level of FD inhibition required for reducing cell lysis by 90% is much higher at ∼99%, pushing the SM affinity estimated to ∼0.01 μM, that is, 10-fold higher for reducing disease severity (Figure 13B).

The drug affinity requirements for complement targets vary significantly due to differences in half-lives, concentrations, and competition of drug binding with other proteins in the complement pathway. For SM modality, complement proteins C3, FB, C5, C6, and FD required affinities around 10–100 nM for 90% TE, but affinity required for 90% reduction in cell lysis was almost an order of magnitude more potent for C5, FD, and C6 (Figure 13C). Additionally, C3 and C5-convertases needed more potent sub-nanomolar affinity SM compounds for both TE and cell lysis.

For LM-Ab modality, C3/C5-convertases, C5, and C7 are on the lower end of the drug affinity range of ≤1 nM for 90% TE (Figure 13D). But the affinity needed for cell lysis *via* C7 inhibition is 100 times more potent than the affinity required for just 90% TE indicating C7 inhibition of 99.9%, or more is required to reduce disease severity. C3bBclose/open forms need ∼0.1 p.m. affinity for TE; however, the impact of cell lysis seems to be prominent at even lower affinities for the “closed” form of C3bB.

## 4 Discussion

A comprehensive QSP model describing complement activation through alternative and terminal pathways has been developed in this work. The complement model differentiates between the pathway activation dynamics in plasma and on the cell surface. The model also includes a detailed description of several plasma and cell-surface regulatory proteins such as Properdin, clusterin, vitronectin, CD59, CR1, DAF, FH, and FI. The effect of Properdin has been described on the stabilization of the C3 and C5-convertase, on promoting the association of C3b to FB as well as its binding to the surface-bound C3b or other ligands, and using the unoccupied Properdin oligomer-binding sites as receptors for nascent C3b and preformed C3-proconvertase/convertase. This work provides, to date, the most comprehensive mathematical implementation for Properdin stoichiometry and its effect on alternative pathway activation based on available experimental evidence.

The complement model was validated using published or in-house GSK data from *in vitro* assays of alternative/terminal pathways and patient data on disease states. A “unit testing” approach was utilized where separate validation was done for different components of the complex complement model such as alternative pathway, terminal pathway, fluid-phase tickover, a closed *in vitro* system with cells, and overall *in vivo* dynamics. This provided an integrated computational and experimental workflow for the utilization of a variety of preclinical and clinical data for model predictions. It also allowed better management of model complexity and higher confidence in the predictions of the combined model as several datasets were used for calibration and validation of model dynamics.

To assess the effect of drug targets in disease pathologies, a reduction in FH and CD59 was used as a proximate mechanistic description of complement activation in aHUS and PNH, respectively. The effect of complement targets on downstream biomarkers and cell lysis was assessed for these simulated disease states in the QSP model. One limitation of the complement model developed in this study is that it considers the lysis of RBCs only, but in autoimmune diseases such as aHUS, other host cells such as platelets would also be lysed in additional to RBCs (Raina et al., 2019). Nevertheless, model simulations provide a preliminary way of understanding the effect of deficiency of complement proteins, their role in autoimmune diseases, and potential treatments to reduce complement activation. Detailed mechanistic models for a more exact assessment of specific complement-driven diseases can be developed in the future.

For reducing the complement activation due to CD59 reduction in aHUS, 90% inhibition of C3, FB, FD, C5, C6, FP, C3b, C3Bb, and C3-convertase (C3bBb and C3bBbP) was predicted to lead to a significant reduction in cell lysis. For inhibiting complement activation due to FH deficiency, drug compounds targeting C3, FB, C5, FP, C3b, C3bB, and C3-convertase at 90% inhibition are predicted to be efficacious in reducing disease severity. Several of these complement proteins, such as C5, FB, FD, C6, and FP, have either approved treatments targeting them (e.g., anti-C5 eculizumab) or drugs in preclinical or clinical stages of development (Mastellos et al., 2019; Zelek et al., 2019). The modeling results further highlighted the complement fragments or intermediates such as C3b, C3bB, and C3-convertases as potential targets for drug development. These assessments were extensively used to support target validation for complement programs in GSK and provided an early assessment of efficacy for improving severity in complement-driven diseases.

A few other targets were also predicted to inhibit complement activation driven by CD59 and FH deficiency, however at a sustained target engagement requirement of 99% inhibition which posed uncertainty around feasibility of dosing high drug amounts to maintain high target inhibitions. Thus, an assessment of the dosing requirements for complement proteins using small/large-molecule modalities was also warranted. Small- and large-molecule Ab compounds, with PK properties that lie within the feasible range for these modalities, were simulated to provide estimates for the feasibility of dosing with these modalities. These assessments were initiated around the target validation phase of drug development to support target selection based on not only the reduction in disease severity but also dosing feasibility. The modeling results were also used to inform lead discovery efforts for pursuing the right modality for a target.

Key trends emerged from the QSP assessment, which were used to guide the selection of target–modality pairs for drug development. Complement proteins that are present in very high concentrations or have high synthesis/turnover rates (e.g., C3, FB, FD, and C6) are challenging to block in the plasma with LM-Ab compounds as the drug doses in molar amounts needed for target engagement compared with the total target would be higher than their feasible dose range (≤20 mg/kg). Because of high dosing requirements for target inhibition in plasma, strategies for localized dosing in the tissues, for example, intravitreous delivery of FD-targeting mAb lampalizumab in the eye (Mastellos et al., 2019), have been adopted. However, lampalizumab failed to achieve its primary endpoint in Phase 3 clinical trials for age-related macular degeneration (AMD) (Holz et al., 2018), and the specific reasons for failure due to drug bioavailability or efficacy are yet to be addressed.

The complement model simulations also predict that SM compounds are ideal for maintaining sustained target engagement for high concentration complement proteins due to the low molecular weights and high molar amounts per mg of SM doses. However, the complement proteins/fragments that have a short half-life (e.g., C3b, C3bB, and C3bBb) were predicted to be challenging or infeasible to engage with SM compounds due to a high drug affinity requirement to “catch” the target before consumption in the pathway. LM-Ab compounds which have higher affinities were able to engage these targets within their feasible dose ranges and are ideal candidates for targeting short-lived complement fragments or complexes.

Another limitation of this study is that the early dose estimates are based on “typical” PK and affinities for drug modalities. These preliminary estimates should be refined during lead optimization as the specific PK properties of drug candidates become available. Modalities such as peptides were not assessed here for simplicity; however, the principles discussed in this work can be applied for the assessment of several modalities. Also, only systemic dosing was considered; however, it may be feasible to attain target engagement in a specific organ/tissue with localized dosing even though significant target engagement overall in plasma may not be feasible due to high target amounts.

In addition to feasible doses for modality selection, another aspect explored in this work was the estimation of drug affinities needed for engaging the complement proteins. During lead optimization, attaining the right level of drug affinity is extremely critical for success in later studies to demonstrate the required level of target engagement. In addition, the drug affinity requirements for 90% target engagement (TE) may be different from affinity requirements for 90% reduction in disease severity if the level of TE required for disease modulation is much higher or lower than the usually targeted 90% level. Thus, an assessment of affinity requirements for TE and cell lysis was done separately, and significant differences in the affinity requirements were observed for targets. For SM modality, complement proteins C3, FB, C5, C6, and FD required affinities around 10–100 nM for 90% TE, but the K_D_ required for 90% reduction in cell lysis was almost an order of magnitude higher for C5, FD, and C6. Similarly, for LM-Ab modality, drug affinity requirements for C7 are on the lower end of the drug affinity range of ≤1 nM for 90% TE, but the K_D_ needed for cell lysis is 100 times more potent. These analyses indicate the importance for accounting for both TE and biomarker/severity endpoints for affinity predictions at the lead optimization stage to prevent the termination of the compounds in later stages of development and improve their probability of success.

The kinetic parameters in the complement model have been set based on literature references, and for parameters without direct literature evidence, the values were assumed based on similar biological processes or proteins. An assessment of the impact of parameter variability in the QSP model using virtual patients and virtual populations (Klinke, 2008; Schmidt et al., 2013; Allen et al., 2016) is beyond the scope of the present assessment and will be covered in future publications. The focus in this work was to provide guidelines for target validation and lead optimization of complement targets and related modalities in early discovery rather than a precise estimate of variability in clinical responses from modulating complement proteins. Thus, assessments have focused on simulating proximate disease states for aHUS and PNH for potential complement targets and doses/affinities for common drug modalities. A detailed assessment of variability in response to biological patient variability and clinical response will be part of further work using the complement model.

## Data Availability

The raw data supporting the conclusion of this article will be made available by the authors, without undue reservation.

## Abbreviations

aHUS, atypical hemolytic uremic syndrome; AP, alternative pathway; C34b*, cell surface binding site; Cn, clusterin; CR1, complement receptor type 1; DAF, decay accelerating factor or CD55; FB, factor B; FD, factor D; FH, factor H; FI, factor I; FP, factor P or Properdin; FPn, Properdin oligomer; GPE, guinea pig erythrocytes; K_D_, drug affinity; LM-Ab, large-molecule antibody; MAC, membrane attack complex; P*, Properdin binding site; PNH, paroxysmal nocturnal hemoglobinuria; QSP, quantitative systems pharmacology; SA, sialic acid; SLE, systemic lupus erythematosus; SM, small molecule; TE, target engagement; TMDD, target-mediated drug disposition; ULQ, upper limit of quantitation; Vd, volume of distribution; Vn, vitronectin.
