# Supplementary material for: Mathematical Modeling of Complement Pathway Dynamics for Target Validation and Selection of Drug Modalities for Complement Therapies
Source: Front Pharmacol. 2022 Apr 19;13:855743. doi: 10.3389/fphar.2022.855743 (PMC9061988; doi:10.3389/fphar.2022.855743)
Supplement: Supplementary file 2 [file Table1.DOCX]

**Parameter References**

[1] M. K. Pangburn, R. D. Schreiber, and H. J. Müller-Eberhard, “Formation of the initial C3 convertase of the alternative complement pathway. Acquisition of C3b-like activities by spontaneous hydrolysis of the putative thioester in native C3.,” *The Journal of experimental medicine*, vol. 154, no. 3, pp. 856–867, 1981.

[2] E. L. Pryzdial and D. E. Isenman, “A thermodynamic study of the interaction between human complement components C3b or C3(H2O) and factor B in solution.,” *J. Biol. Chem.*, vol. 263, no. 4, pp. 1733–1738, Feb. 1988.

[3] M. K. Pangburn and H. J. Müller-Eberhard, “The C3 convertase of the alternative pathway of human complement. Enzymic properties of the bimolecular proteinase.,” *Biochem J*, vol. 235, no. 3, pp. 723–730, May 1986.

[4] N. Rawal and M. K. Pangburn, “Functional Role of the Noncatalytic Subunit of Complement C5 Convertase,” *J Immunol*, vol. 164, no. 3, pp. 1379–1385, Feb. 2000, doi: 10.4049/jimmunol.164.3.1379.

[5] R. B. Sim, T. M. Twose, D. S. Paterson, and E. Sim, “The covalent-binding reaction of complement component C3.,” *Biochem. J*, vol. 193, pp. 115–127, 1981.

[6] D. E. Hourcade and L. M. Mitchell, “Access to the Complement Factor B Scissile Bond Is Facilitated by Association of Factor B with C3b Protein,” *Journal of Biological Chemistry*, vol. 286, no. 41, pp. 35725–35732, Oct. 2011, doi: 10.1074/jbc.M111.263418.

[7] C. L. Harris, R. J. M. Abbott, R. A. Smith, B. P. Morgan, and S. M. Lea, “Molecular Dissection of Interactions between Components of the Alternative Pathway of Complement and Decay Accelerating Factor (CD55),” *Journal of Biological Chemistry*, vol. 280, no. 4, pp. 2569–2578, Jan. 2005, doi: 10.1074/jbc.M410179200.

[8] M. D. Kazatchkine, D. T. Fearon, and K. F. Austen, “Human Alternative Complement Pathway: Membrane-Associated Sialic Acid Regulates the Competition between B and β1H for Cell-Bound C3b,” *J Immunol*, vol. 122, no. 1, pp. 75–81, Jan. 1979.

[9] R. G. DiScipio, “The binding of human complement proteins C5, factor B, β1H and properdin to complement fragment C3b on zymosan,” *Biochemical Journal*, vol. 199, no. 3, pp. 485–496, Dec. 1981, doi: 10.1042/bj1990485.

[10] A. A. Korotaevskiy, L. G. Hanin, and M. A. Khanin, “Non-linear dynamics of the complement system activation,” *Mathematical Biosciences*, vol. 222, no. 2, pp. 127–143, Dec. 2009, doi: 10.1016/j.mbs.2009.10.003.

[11] F. Forneris *et al.*, “Structures of C3b in Complex with Factors B and D Give Insight into Complement Convertase Formation,” *Science*, vol. 330, no. 6012, pp. 1816–1820, Dec. 2010, doi: 10.1126/science.1195821.

[12] M. V. L. Campagne and C. Wiesmann, “Co-crystal structure of factor D and anti-factor D antibody,” US20110165648 A1, Jul. 07, 2011.

[13] K. J. Katschke *et al.*, “Inhibiting Alternative Pathway Complement Activation by Targeting the Factor D Exosite,” *J. Biol. Chem.*, vol. 287, no. 16, pp. 12886–12892, Apr. 2012, doi: 10.1074/jbc.M112.345082.

[14] M. K. Pangburn and H. J. Mueller-Eberhard, “Kinetic and thermodynamic analysis of the control of C3b by the complement regulatory proteins factors H and I,” *Biochemistry*, vol. 22, no. 1, pp. 178–185, Jan. 1983, doi: 10.1021/bi00270a026.

[15] J. Bernet, J. Mullick, Y. Panse, P. B. Parab, and A. Sahu, “Kinetic Analysis of the Interactions between Vaccinia Virus Complement Control Protein and Human Complement Proteins C3b and C4b,” *J. Virol.*, vol. 78, no. 17, pp. 9446–9457, Sep. 2004, doi: 10.1128/JVI.78.17.9446-9457.2004.

[16] C. Q. Schmidt *et al.*, “A New Map of Glycosaminoglycan and C3b Binding Sites on Factor H,” *J Immunol*, vol. 181, no. 4, pp. 2610–2619, Aug. 2008, doi: 10.4049/jimmunol.181.4.2610.

[17] F. Bexborn, P. O. Andersson, H. Chen, B. Nilsson, and K. N. Ekdahl, “The tick-over theory revisited: Formation and regulation of the soluble alternative complement C3 convertase (C3(H2O)Bb),” *Molecular Immunology*, vol. 45, no. 8, pp. 2370–2379, Apr. 2008, doi: 10.1016/j.molimm.2007.11.003.

[18] D. E. Hourcade, “The Role of Properdin in the Assembly of the Alternative Pathway C3 Convertases of Complement,” *J. Biol. Chem.*, vol. 281, no. 4, pp. 2128–2132, Jan. 2006, doi: 10.1074/jbc.M508928200.

[19] J. M. Weiler, M. R. Daha, K. F. Austen, and D. T. Fearon, “Control of the amplification convertase of complement by the plasma protein beta1H.,” *Proc Natl Acad Sci U S A*, vol. 73, no. 9, pp. 3268–3272, Sep. 1976.

[20] S. H. M. Rooijakkers *et al.*, “Structural and functional implications of the alternative complement pathway C3 convertase stabilized by a staphylococcal inhibitor,” *Nat Immunol*, vol. 10, no. 7, Art. no. 7, Jul. 2009, doi: 10.1038/ni.1756.

[21] T. Seya, V. M. Holers, and J. P. Atkinson, “Purification and functional analysis of the polymorphic variants of the C3b/C4b receptor (CR1) and comparison with H, C4b-binding protein (C4bp), and decay accelerating factor (DAF).,” *J Immunol*, vol. 135, no. 4, pp. 2661–2667, Oct. 1985.

[22] N. Rawal and M. K. Pangburn, “Formation of High-Affinity C5 Convertases of the Alternative Pathway of Complement,” *J Immunol*, vol. 166, no. 4, pp. 2635–2642, Feb. 2001, doi: 10.4049/jimmunol.166.4.2635.

[23] E. R. Podack, G. Biesecker, W. P. Kolb, and H. J. Müller-Eberhard, “The C5b-6 Complex: Reaction with C7, C8, C9,” *J Immunol*, vol. 121, no. 2, pp. 484–490, Aug. 1978.

[24] C. K. N. Li and R. P. Levine, “Rate process in the final stage of complement hemolysis,” *Immunochemistry*, vol. 14, no. 6, pp. 421–428, Jun. 1977, doi: 10.1016/0019-2791(77)90167-7.

[25] S. Meri *et al.*, “Human protectin (CD59), an 18,000-20,000 MW complement lysis restricting factor, inhibits C5b-8 catalysed insertion of C9 into lipid bilayers.,” *Immunology*, vol. 71, no. 1, pp. 1–9, Sep. 1990.

[26] T. Kinoshita, M. E. Medof, R. Silber, and V. Nussenzweig, “Distribution of decay-accelerating factor in the peripheral blood of normal individuals and patients with paroxysmal nocturnal hemoglobinuria.,” *J Exp Med*, vol. 162, no. 1, pp. 75–92, Jul. 1985, doi: 10.1084/jem.162.1.75.

[27] G. D. Ross *et al.*, “Disease-associated loss of erythrocyte complement receptors (CR1, C3b receptors) in patients with systemic lupus erythematosus and other diseases involving autoantibodies and/or complement activation.,” *J Immunol*, vol. 135, no. 3, pp. 2005–2014, Sep. 1985.

[28] A. J. Sliwinski and N. J. Zvaifler, “Decreased synthesis of the third component of complement (C3) in hypocomplementenic systemic lupus erythematosus,” *Clin Exp Immunol*, vol. 11, no. 1, pp. 21–29, May 1972.

[29] C. B. Carpenter, S. Ruddy, I. H. Shehadeh, H. J. Müller-Eberhard, J. P. Merrill, and K. F. Austen, “Complement metabolism in man: hypercatabolism of the fourth (C4) and third (C3) components in patients with renal allograft rejection and hereditary angioedema (HAE),” *J Clin Invest*, vol. 48, no. 8, pp. 1495–1505, Aug. 1969.

[30] C. A. Alper and F. S. Rosen, “Metabolism of radiolabelled complement proteins in health and disease,” *SpringerLink*, pp. 195–220, 1984, doi: 10.1007/978-1-349-06680-3_10.

[31] J. Schaller, S. Gerber, U. Kaempfer, S. Lejon, and C. Trachsel, *Human Blood Plasma Proteins: Structure and Function*. John Wiley & Sons, 2008.

[32] M. Pascual, G. Steiger, J. Estreicher, K. Macon, J. E. Volanakis, and J. A. Schifferli, “Metabolism of complement factor D in renal failure,” *Kidney International*, vol. 34, no. 4, pp. 529–536, Oct. 1988, doi: 10.1038/ki.1988.214.

[33] C. Licht *et al.*, “Successful plasma therapy for atypical hemolytic uremic syndrome caused by factor H deficiency owing to a novel mutation in the complement cofactor protein domain 15,” *American Journal of Kidney Diseases*, vol. 45, no. 2, pp. 415–421, Feb. 2005, doi: 10.1053/j.ajkd.2004.10.018.

[34] J. Møller Rasmussen *et al.*, “Three cases of factor I deficiency: the effect of treatment with plasma.,” *Clin Exp Immunol*, vol. 74, no. 1, pp. 131–136, Oct. 1988.

[35] J. D. Greenstein, P. W. Peake, and J. A. Charlesworth, “The kinetics and distribution of C9 and SC5b-9 in vivo: effects of complement activation,” *Clinical & Experimental Immunology*, vol. 100, no. 1, pp. 40–46, Apr. 1995, doi: 10.1111/j.1365-2249.1995.tb03601.x.

[36] N. R. Cooper and H. J. Müller-Eberhard, “The Reaction Mechanism of Human C5 in Immune Hemolysis,” *J Exp Med*, vol. 132, no. 4, pp. 775–793, Oct. 1970.

[37] T. E. Mollnes, T. S. Jokiranta, L. Truedsson, B. Nilsson, S. Rodriguez de Cordoba, and M. Kirschfink, “Complement analysis in the 21st century,” *Molecular Immunology*, vol. 44, no. 16, pp. 3838–3849, Sep. 2007, doi: 10.1016/j.molimm.2007.06.150.

[38] M. Oppermann and O. Götze, “Plasma clearance of the human C5a anaphylatoxin by binding to leucocyte C5a receptors,” *Immunology*, vol. 82, no. 4, pp. 516–521, Aug. 1994.

[39] M. Oppermann and O. Götze, “Characterization of physiologic breakdown products of the complement fragment Ba,” *Molecular Immunology*, vol. 31, no. 4, pp. 307–314, Mar. 1994, doi: 10.1016/0161-5890(94)90128-7.

[40] E. Sim, A. B. Wood, L.-M. Hsiung, and R. B. Sim, “Pattern of degradation of human complement fragment, C3b,” *FEBS Letters*, vol. 132, no. 1, pp. 55–60, 1981, doi: 10.1016/0014-5793(81)80426-7.

[41] K. Iida, R. Mornaghi, and V. Nussenzweig, “Complement receptor (CR1) deficiency in erythrocytes from patients with systemic lupus erythematosus.,” *J Exp Med*, vol. 155, no. 5, pp. 1427–1438, May 1982, doi: 10.1084/jem.155.5.1427.
