# Supplementary material for: Mathematical Modeling of Complement Pathway Dynamics for Target Validation and Selection of Drug Modalities for Complement Therapies
Source: Front Pharmacol. 2022 Apr 19;13:855743. doi: 10.3389/fphar.2022.855743 (PMC9061988; doi:10.3389/fphar.2022.855743)
Supplement: Supplementary file 4 [file Table2.DOCX]

**iC3b Quantitative ELISA Assay**

**Abbreviations**

mAb - Monoclonal Antibody

pAb - Polyclonal Antibody

ab - Antibody

α-iC3b mAb c4 - anti-human iC3b antibody clone 4

α-Ab - anti-human antibody

NHS - Normal Human Serum

TMB - 3,3′,5,5′-Tetramethylbenzidine

BSA - Bovine Serum Albumin

PBS - Phosphate Buffered Saline

RT - Room Temperature

Min - Minutes

Hr - Hours

FAC - Final Assay Concentration

**Reagents**

| Reagents/Equipment | Supplier/Manufacturer | Order Nos. | B Batch | C Conc |
| --- | --- | --- | --- | --- |
| Mouse (IgG_2a_) anti-human C3 Clone bH6 | Hycult | HM2168 | 05113M0106-B | 0.82mg/ml |
| Rat (IgG_1_) anti-human C3dg/iC3b/C3g Clone 9 | CambridgeBioscience  /Hycult | HM2199 | 1206M1206-E | 0.1mg/ml |
| Human iC3b (Protein Analyte) | Complement Technologies | A115 | Lot 10 | 1.08mg/ml |
| Mouse anti –Rat IgG_1_ – HRP conjugate | Southern Biotech | 3061-05 | F0111-x101c | unknown |
|  |  |  |  |  |
| TMB substrate | SIGMA | N301 |  |  |
| BSA | SIGMA | A4503 |  |  |
| PBS | Gibco | 14040 |  |  |
| 1M HCl | SIGMA | 35328-1L |  |  |

Buffers

ELISA Wash Solution - MilliQ PBS with 0.05% (v/v) Tween20

Assay Buffer - PBS/0.05% (v/v) Tween20 plus 1% BSA

Sample Buffer - PBS/0.05% (v/v) Tween20 plus 10mM EDTA

ELISA Blocking Buffer - 2% BSA in PBS

Stopping reagent - 1M HCl

**Methods** (Also see plate maps)

**Day 1**

**Plate Preparation**

1. Coat required number of NUNC MaxiSorp^®^ flat bottom 96 well plate(s) with 75μl/well of 5ug/ml Hycult anti neo-iC3b Clone bH6 in PBS according to the plate map and reagent preparation.
2. Seal the plate(s) and incubate at 4degC for at least 15hrs.

**Day 2**

**Preparation of Samples**

All samples were prepared on the day of the experiment and kept on ice until required.

Samples were diluted in sample buffer according to the reagent preparation and plate maps

**ELISA METHOD**

*All the following incubation steps are performed on a Heidolph Titramax 1000 plate shake at 650-700rpm*

1. Block the plate(s) with 150μl/well of ELISA blocking buffer and incubate at RT for 1.0 hrs
2. Wash the plate(s) with MilliQ PBS plus 0.05% (v/v) Tween20, using the plate washer

(BioTek Plate Washer, 3x wash setting, prog. 17).

1. Make up the required volume(s) of the iC3b antigen to form a 3 fold standard curve, in sample buffer, at the required concentrations and in accordance with the plate map and reagent preparation. Add 75ul/well of iC3bstandards and diluted samples according to the plate map plan.
2. Seal the plate(s) and incubate at RT for 2hr.
3. Wash the plate(s) with MilliQ PBS plus 0.05% (v/v) Tween20, using the plate washer (BioTek Plate Washer, 3x wash setting, ELISA WASH prog. 17).
4. Dilute ‘detection’ antibodies to the required concentrations, in ELISA buffer, as described in the reagent preparations and add 80μl/well in accordance with the plate map.
5. Seal the plate and incubate at RT for 1hr.
6. After incubation, repeat the wash step as described in step 5.
7. Dilute the anti rat ‘labelling’ antibody to the required dilution, in ELISA buffer, as described in the reagent preparations and add 70μl/well in accordance with the plate map.
8. Seal the plate and incubate at RT for 60mins.
9. After incubation, repeat the wash step as described in step 5.
10. Add 100μl/well TMB in accordance with the plate map
11. Leave to develop for ~5-15 minutes until colour appears to be at an appropriate intensity.
12. Add 50μl/well 1M HCl to end development
13. Read plates at 450nm on the Biotek EPOCH plate reader (with 570nm signal subtracted for background) and save data.
